# Predicting Dose-Dependent Carcinogenicity of Chemical Mixtures Using a Novel Hybrid Neural Network Framework and Mathematical Approach

**DOI:** 10.3390/toxics11070605

**Published:** 2023-07-12

**Authors:** Sarita Limbu, Sivanesan Dakshanamurthy

**Affiliations:** Lombardi Comprehensive Cancer Center, Georgetown University Medical Center, Washington, DC 20057, USA

**Keywords:** hybrid neural network, dose-dependent carcinogenicity, chemical mixtures, machine learning, per- and polyfluorinated substances

## Abstract

This study addresses the challenge of assessing the carcinogenic potential of hazardous chemical mixtures, such as per- and polyfluorinated substances (PFASs), which are known to contribute significantly to cancer development. Here, we propose a novel framework called HNN_MixCancer_ that utilizes a hybrid neural network (HNN) integrated into a machine-learning framework. This framework incorporates a mathematical model to simulate chemical mixtures, enabling the creation of classification models for binary (carcinogenic or noncarcinogenic) and multiclass classification (categorical carcinogenicity) and regression (carcinogenic potency). Through extensive experimentation, we demonstrate that our HNN model outperforms other methodologies, including random forest, bootstrap aggregating, adaptive boosting, support vector regressor, gradient boosting, kernel ridge, decision tree with AdaBoost, and KNeighbors, achieving a superior accuracy of 92.7% in binary classification. To address the limited availability of experimental data and enrich the training data, we generate an assumption-based virtual library of chemical mixtures using a known carcinogenic and noncarcinogenic single chemical for all the classification models. Remarkably, in this case, all methods achieve accuracies exceeding 98% for binary classification. In external validation tests, our HNN method achieves the highest accuracy of 80.5%. Furthermore, in multiclass classification, the HNN demonstrates an overall accuracy of 96.3%, outperforming RF, Bagging, and AdaBoost, which achieved 91.4%, 91.7%, and 80.2%, respectively. In regression models, HNN, RF, SVR, GB, KR, DT with AdaBoost, and KN achieved average R^2^ values of 0.96, 0.90, 0.77, 0.94, 0.96, 0.96, and 0.97, respectively, showcasing their effectiveness in predicting the concentration at which a chemical mixture becomes carcinogenic. Our method exhibits exceptional predictive power in prioritizing carcinogenic chemical mixtures, even when relying on assumption-based mixtures. This capability is particularly valuable for toxicology studies that lack experimental data on the carcinogenicity and toxicity of chemical mixtures. To our knowledge, this study introduces the first method for predicting the carcinogenic potential of chemical mixtures. The HNN_MixCancer_ framework offers a novel alternative for dose-dependent carcinogen prediction. Ongoing efforts involve implementing the HNN method to predict mixture toxicity and expanding the application of HNN_MixCancer_ to include multiple mixtures such as PFAS mixtures and co-occurring chemicals.

## 1. Introduction

Identifying carcinogenic chemicals is crucial for human health, as they present a severe toxicological risk that can lead to adverse health effects, including cancer development. Carcinogens are substances that have the potential to initiate or promote the development of cancerous cells in living organisms. The standard method for assessing the carcinogenic potential of chemicals is the 2 year rodent carcinogenicity assay, which has been used for over 50 years [1]. This assay involves exposing rodents to various doses of a chemical over an extended period and monitoring for the development of tumors. While this method has provided valuable insights into the carcinogenicity of numerous substances, these long-term animal studies are resource-intensive, time-consuming, and ethically challenging. Moreover, environmental exposure to carcinogenic chemicals rarely occurs in isolation but rather involves complex mixtures. These mixtures consist of various manmade contaminants that pervade the air we breathe, the water we drink, the soil in which we grow our food, and the food we consume, leading to adverse health effects, including cancer. Notable examples of these contaminants include pesticides, per- and polyfluorinated substances (PFASs), polycyclic aromatic hydrocarbons (PAHs), metals, and polychlorinated biphenyls (PCBs). These substances find their way into the environment through industrial processes, waste disposal, and the use of certain consumer products. As a result, individuals are exposed to a multitude of chemicals simultaneously, increasing the complexity of evaluating their carcinogenic potential.

The assessment of chemical mixture carcinogenicity is hindered by the vast number of combinations, resource-intensive experiments, and the absence of computational methods, resulting in limited data on the carcinogenicity of chemical mixtures. Furthermore, assessing the carcinogenic chemical mixtures is a formidable task due to the added intricacy introduced by varying concentrations of the individual chemical components. Each chemical within a mixture can interact with other chemicals, potentially amplifying or attenuating their carcinogenic effects. Determining the precise contribution of each component to the overall carcinogenic potential becomes challenging, requiring sophisticated experimental designs and analytical techniques. Typically, the carcinogenic potential of chemical combinations is assessed, focusing on whether one chemical promotes the carcinogenicity initiated by another chemical, even if the second chemical, in isolation, does not exhibit carcinogenic properties at carcinogenic doses or different concentrations [2,3,4]. Animal models are commonly used for such assessments, wherein animals are exposed to specific combinations of chemicals to observe their collective effects on tumor development and progression. Moreover, in-vitro cell line models have been utilized to study the combined carcinogenic effects of chemicals. Studies have demonstrated synergistic effects on DNA adduct formation when phthalates, a group of chemicals commonly found in plastics and personal care products, are co-exposed in these cell line models [5,6]. These findings highlight the importance of considering chemical interactions within mixtures and their potential to enhance carcinogenic outcomes. Additionally, research has investigated the synergistic and combined anticancer effects of anticancer drugs in animal models and cell lines to combat carcinogenicity [7,8,9]. To support the current study, an extensive collection of literature references on the combined effects of carcinogens and noncarcinogens in chemical mixtures is utilized in this study [10,11,12,13,14,15,16,17,18,19,20,21,22,23,24,25,26,27,28,29,30,31,32,33,34,35,36,37,38,39,40,41,42,43,44,45,46,47,48,49,50,51,52,53,54,55,56,57,58,59,60,61,62,63] (see Supplementary Materials).

The computational method offers a highly efficient and cost-effective alternative to traditional experimental approaches for assessing the carcinogenic potential of chemical mixtures. While the concentration addition (CA) model has been widely utilized to calculate the toxicity of binary mixtures [64,65], its application in evaluating the carcinogenicity of mixtures has been somewhat limited. In an effort to overcome this limitation, Walker et al. conducted a study employing the dioxin toxic equivalency factor (TEF) approach to determine the dose-additive carcinogenicity of three compound mixtures of dioxins [66]. The TEF approach allows for the conversion of different dioxin congeners into a common unit of toxicity, enabling the calculation of an equitoxic ratio for each compound in the mixture. By employing equal ratios of toxic equivalency (TEQs) for each compound, they aimed to represent an equitoxic ratio in their assessment of the mixture’s carcinogenic potential. However, a significant challenge encountered when utilizing computational methods to evaluate mixture carcinogenicity is the lack of available information regarding the specific carcinogenic concentrations of classified carcinogens. Identifying the precise concentrations at which individual chemicals exhibit carcinogenic effects is crucial for accurate assessments. Unfortunately, such data are often unavailable or incomplete, impeding the comprehensive evaluation of mixture carcinogenicity using computational approaches. To address these challenges and improve the accuracy of mixture carcinogenicity assessments, further research is needed to expand the application of computational models beyond simple single chemical simulations or the simple application of quantitative structure–activity relationship (QSAR) methods for chemical mixtures. This includes developing and refining approaches that account for varying levels of carcinogenic potency among mixture components and integrating a vast number of mixture data to enhance the predictive capabilities of computational models.

Traditional experimental methods for assessing carcinogenicity in animal models face significant limitations due to the vast number of chemical combinations and the wide range of doses associated with each chemical within a mixture. Furthermore, the lack of computational methodologies capable of evaluating the carcinogenic effects of chemical mixtures exacerbates this challenge. Consequently, it is crucial to develop innovative approaches to gain a more comprehensive understanding of the risks posed by both known and unknown mixtures. This challenge is particularly evident in the case of PFASs, a class of chemicals known for their persistent nature, the tendency to exist as mixtures, and the potential to induce cancer. Recognizing this gap, the primary objective of our study is to introduce a novel methodology that addresses these challenges by predicting the dose-dependent carcinogenic potential of chemical mixtures while simultaneously enriching the dataset on carcinogenic mixtures. To accomplish this, in addition to using the literature-reported carcinogenic mixtures, we expand the mixtures dataset by generating assumption-based virtual chemical mixtures for all the classification models. Assumptions are based on the existing carcinogenic and noncarcinogenic mixtures (see details in Section 2). By incorporating these virtual mixtures, we aim to supplement the limited dataset and provide a more comprehensive representation of chemical mixtures. This dataset augmentation allows for a broader exploration of various mixture compositions and concentrations, enabling more robust predictions of carcinogenicity.

In our study, we develop an innovative hybrid neural network method called ‘HNN-MixCancer’ specifically designed to predict the carcinogenic potential of various chemical binary mixtures accurately. This method combines the strengths of neural network algorithms with a mathematical framework that models the unique characteristics of chemical mixtures. By leveraging the complex relationships between mixture components and their concentrations modeled by a mathematical framework, the HNN_MixCancer_ provides reliable predictions on the carcinogenicity of these mixtures. To ensure accuracy, we construct classification models within the HNN-MixCancer framework, considering factors such as the chemical composition and concentrations of the mixtures. Additionally, we enhance the training data by introducing assumption-based virtual chemical mixtures. Preliminary results from the HNN_MixCancer_ demonstrate promising outcomes, successfully predicting the dose-dependent carcinogenic potential of chemical mixtures. Rigorous testing and validation processes against known experimental data are conducted to evaluate the model’s performance, ensuring its reliability and accuracy in predicting the carcinogenic properties of diverse chemical mixtures.

The HNN_MixCancer_ opens up new avenues for predicting the carcinogenic potential of chemical mixtures in terms of carcinogenic or noncarcinogenic, categorical carcinogenicity, and carcinogenic potency, offering a valuable tool for risk assessment and decision-making in various industries and regulatory bodies. The introduction of this novel methodology, alongside the enhanced dataset on carcinogenic mixtures, represents a significant advancement in computational toxicology. Furthermore, the method developed in this study lays the foundation for future research endeavors, inspiring further innovation and exploration of new approach methodologies (NAMs) in profiling mixture carcinogenesis alternatives to animal models.

## 2. Methods

### 2.1. Data Collection

#### 2.1.1. Mixtures from the Literature and Drug Combination Database (DCDB)

##### Literature-Derived Mixtures

We obtained 54 carcinogenic binary chemical mixtures, including PFAS (Supplementary Table S1), and 25 binary drug mixtures without carcinogenic effects but exhibiting anticancer properties (Supplementary Table S2) from various literature sources.

##### Mixtures from the Drug Combination Database (DCDB)

We obtained 942 drug combinations from the DCDB database at http://public.synergylab.cn/dcdb/download.jsf (accessed on 20 June 2021). Out of these combinations, 757 binary combinations were mapped with the corresponding drug bank ID using the DCC_ID of each drug. Mordred descriptors were calculated for a subset of 646 drug combinations. The SMILES and drug bank ID information for the DCDB drug combinations were sourced from www.drugbank.ca.

##### Literature-Derived and DCDB Mixture Data for Binary Classification Models

We gathered 54 carcinogenic and 25 noncarcinogenic chemical combinations from the existing literature, along with 30 drug combinations from DCDB identified as noncarcinogens. Due to the absence of precise dose values in this dataset, we assumed equal concentrations of the chemical components in each mixture when calculating descriptors.

##### Validating Binary Classification Models with Assumption-Based Virtual Mixture Dataset

To test the accuracy of the prediction models developed for assumption-based virtual binary mixtures, we utilized a validation set comprising 54 carcinogenic and 25 noncarcinogenic binary mixtures collected from the literature (Section 1) and 30 binary drug combinations from the DCDB. The procedure for constructing the assumption-based virtual binary mixtures is described in Section 2.

#### 2.1.2. Compilation of Mixture Data for the Creation of an Assumption-Based Virtual Mixture Dataset

Due to the limited availability of experimental data on the carcinogenicity of chemical mixtures for the purpose of developing machine learning models, we sought out individual chemicals classified as carcinogenic or noncarcinogenic from reputable data sources. These sources included the Military Exposure Guideline (MEG), the National Toxicology Program (NTP) of the US Department of Health and Human Services, the International Agency for Research on Cancer (IARC), the Japan Society for Occupational Health (JSOH), and the Carcinogenic Potency Database (CPDB). Subsequently, using a series of assumptions outlined above, we then generated mixtures comprising both carcinogenic and noncarcinogenic substances.

##### Chemical Exposure Guidelines for Deployed Military Personnel Version 1.3 (MEG)

The carcinogenicity data were sourced from the Technical Guide 230 Environmental Health Risk Assessment and Chemical Exposure Guidelines for Deployed Military Personnel (MEG) [67]. The MEG dataset encompasses acute and chronic exposure data for carcinogenic air, water, and soil chemicals. These carcinogenic chemicals are classified into five distinct groups: Group A (human carcinogen), Group B (probable human carcinogen), Group C (possible human carcinogen), Group D (not classifiable), and Group E (no evidence of carcinogenicity).

##### National Toxicology Program (NTP)

We collected carcinogenic chemicals classified by the National Toxicology Program (NTP) [68] that fall into two distinct groups: (1) chemicals that are reasonably anticipated to be human carcinogens and (2) chemicals that are known to be human carcinogens.

##### International Agency for Research on Cancer (IARC)

We collected IARC monographs volume 1–125 [69] carcinogenic chemicals that are categorized into five groups: Group 1 (carcinogenic to humans), Group 2A (probably carcinogenic to humans), Group 2B (possibly carcinogenic to humans), Group 3 (not classifiable as to its carcinogenicity to humans), and Group 4 (probably not carcinogenic to humans).

##### The Japan Society for Occupational Health (JSOH)

We collected carcinogenic chemicals from the JSOH (Japan Society for Occupational Health) [70] published Recommendations for Occupational Exposure Limits, which categorize carcinogenic chemicals into three groups: Group 1 (carcinogenic to humans), Group 2A (probably carcinogenic to humans), and Group 2B (possibly carcinogenic to humans).

##### Carcinogenic Potency Database (CPDB)

We obtained the median toxicity dose (TD_50_) rat carcinogenicity data from the CPDB [71] of the NIH. TD_50_ refers to the dose rate in mg/kg body weight/day that, when administered throughout life, induces cancer in half of the test animals. For rat carcinogenicity, we obtained 561 carcinogenic chemicals annotated with TD_50_ values and 605 annotated as noncarcinogenic chemicals.

#### 2.1.3. Generating Virtual Mixtures for Binary Classification Models: Assumptions and Methods

We employed permutations and combinations methods to generate binary and multiple mixture combinations from individual chemicals including emerging PFAS chemicals. Considering the vast number of possible combinations, we used a representative sampling approach to capture the diverse range of combinations while reducing the total number. This was accomplished by selecting chemical samples representing highly similar, medium similar, and low similar (diverse) combinations using similarity metrics such as Tanimoto similarity coefficients [72]. For the purpose of generating virtual binary mixture datasets, we employed various assumptions-based cases to form different combinations of mixtures. These combinations were utilized to determine whether each combination was classified as carcinogenic or noncarcinogenic, with the ultimate goal of attaining the highest level of predictive accuracy. Additionally, we applied the read-across procedure [73], which assumes that similar molecules exhibit similar characteristics, to assign carcinogenic properties. In all of the aforementioned assumptions, we assigned a value of ‘1’ to indicate carcinogenicity for a component chemical or resultant mixture and a value of ‘0’ to indicate a noncarcinogen. For the training and test datasets, we included 80% of the generated data in the training set and the remaining 20% in the test set, following all assumptions.

##### Assumptions Creating the Chemical Mixture Combinations—Case Examples

Case 1: If each chemical in a combination is carcinogenic, and the resultant mixture is considered carcinogenic or toxic (1 + 1 = 1).

Case 2: If each chemical in a combination is noncarcinogenic, and the resultant mixture is considered noncarcinogenic (0 + 0 = 0).

Case 3: If each chemical in a combination is noncarcinogenic, but the resultant mixture is carcinogenic (0 + 0 = 1). In the context of this case 3 assumption scenario for creating a virtual binary mixture, the description of how two chemicals that are noncarcinogenic can result in a carcinogenic mixture is provided in the Supplementary Materials.

Case 4: If at least one chemical in a combination is carcinogenic, and the resultant mixture is considered carcinogenic (1 + 0 = 1). Here, the first chemical is carcinogenic, and the second chemical is noncarcinogenic.

Case 5: If at least one chemical in a combination is carcinogenic, and the resultant mixture is considered carcinogenic (0 + 1 = 1). Here, the first chemical is noncarcinogenic, and the second chemical is carcinogenic.

Similar assumptions were made for other mixture combinations, including multiple mixtures. More explanations of cases 4 and 5 and multiple mixtures are provided in the Supplementary Materials.

##### Considerations of Chemical Dose and Concentration

In this study, we report only the results of the virtual binary mixtures obtained from Case 1 (the combinations were formed by mixing carcinogen with another carcinogen chemical that produces a carcinogenic mixture) and Case 2 (the mixtures were formed by mixing noncarcinogen with another noncarcinogen chemical that produces a carcinogenic combination). Importantly, mixture formation requires concentration information of each component chemical that makes the mixture to calculate the mixture descriptor. Therefore, we used the reported concentration information associated with a chemical while making mixture combinations. In instances where concentration information was unavailable for certain chemical data, we assigned equal concentrations to those cases during the mixture formation process.

Binary chemical classification into carcinogenic and noncarcinogenic categories involved creating virtual mixtures from the following two data sources.

(i) Data source 1 (MEG): Obtained 106 carcinogenic chemicals (Group A, B, and C) and three noncarcinogenic chemicals (Group E) with concentration information. Chemicals in Group D were not considered in either class. We created 5565 carcinogenic binary mixtures from all possible combinations of the 106 carcinogenic single chemicals. We also created three noncarcinogenic binary mixtures from possible combinations of the three noncarcinogenic single chemicals. Then, we calculated the concentration fraction using the concentration of each single chemical and computed the descriptors of the mixtures.
(ii) Data source 5 (CPDB): After removing duplicates and conflicting chemicals, this dataset contributed 508 carcinogenic and 580 noncarcinogenic chemicals compared to data source 1 (MEG). For CPDB data, we calculated the concentration fraction using the TD50 value of each single chemical for carcinogenic mixtures and equal concentration for noncarcinogenic mixtures (as TD50 values do not apply to non-carcinogens). Then, we computed the descriptors of the mixtures.

The training set for the binary classification model included 5565 carcinogenic mixtures from MEG and 5000 randomly selected mixtures (out of 128,778) from CPDB. It also included three noncarcinogenic combinations from MEG and 10,000 randomly selected mixtures (out of 167,910) from CPDB. Thus, the final training set consisted of 10,565 carcinogenic mixtures and 10,003 noncarcinogenic mixtures.

##### Preparation of Distinct Test Dataset: Separating Compounds into Training and Test Set

To make a distinct test dataset, compounds were separated into training and test sets to develop binary classification models, ensuring that the compounds in the test set were not present in the training set.

(i) Data source 1 (MEG): The 106 carcinogenic chemicals were divided into 84 for training and 22 for test sets. From these data, 3486 training and 231 test set carcinogenic binary mixtures were created by considering all possible combinations.
(ii) Data source 5 (CPDB): Among the 508 carcinogens, 408 were allocated for training, and 100 were allocated for the test set. These data created 83,028 training and 4950 test sets carcinogenic binary mixtures by considering all possible combinations. Additionally, the 580 noncarcinogens were divided into 464 for training and 116 for testing. From these data, 107,416 training and 6670 test sets of noncarcinogenic binary mixtures were created by considering all possible binary combinations.

The combined training set for the binary classification model consisted of 10,086 carcinogenic mixtures (3486 from MEG and 6600 randomly selected from the 83,028 in CPDB) and 10,003 noncarcinogenic mixtures (three from MEG and 10,000 randomly selected from the 107,416 in CPDB). The combined test set included 5181 carcinogenic mixtures (231 from MEG and 4950 from CPDB) and 6670 noncarcinogenic mixtures (from CPDB).

#### 2.1.4. Generating Virtual Mixtures for Multiclass Classification Models: Assumptions and Methods

##### Assumptions

1. We focused on intraclass mixtures by combining two chemicals of the same class, resulting in a mixture representing that class. Interclass mixtures are not reported in this study.
2. Mixture formation requires concentration information of each component chemical that makes the mixture to calculate the mixture descriptor. Here, for multiclass classification, the mixtures were formed with equal concentrations of each component chemical to calculate the mixture descriptors.

The multiclass models included three classes: Class 0 for noncarcinogens, Class 1 for possibly carcinogenic and not classifiable chemicals, and Class 2 for carcinogens and probably carcinogens.

More details about the description of the classifications of Class 0, Class 1, and Class 2 are provided in the Supplementary Materials.

The dataset comprised 459 chemicals in Class 0, 604 chemicals in Class 1, and 555 chemicals in Class 2, obtained from five different data sources. Mixtures for each class were created by combining two chemicals from the same class in equal concentrations.

(i) Data 1 (MEG): Chemicals classified into Groups A and B were considered Class 2. Chemicals in Groups C and D were classified as Class 1. Chemicals in Group E were classified as Class 0.
(ii) Data 2 (NTP): Chemicals classified as “reasonably anticipated to be a human carcinogen” or “known to be human carcinogens” were classified as Class 2.
(iii) Data 3 (IARC): Chemicals classified into Groups 1 and 2A were categorized as Class 2 carcinogens, while those in Groups 2B and 3 were classified as Class 1 carcinogens.
(iv) Data 4 (JSOH): Chemicals classified into Groups 1 and 2A were considered Class 2 carcinogens, and those in Group 2B were classified as Class 1 carcinogens.
(v) A total of 882 carcinogenic chemicals were collected from the four data sources mentioned above. The combined dataset consisted of two instances in Class 0, 604 instances in Class 1, and 278 instances in Class 2.
(vi) Data 5 (CPDB): From CPDB chemicals in data source 5, after removing duplicates and repetitive conflicting chemicals, an additional set of 277 carcinogenic chemicals and 457 noncarcinogenic chemicals were obtained. The 277 carcinogenic chemicals were categorized as Class 2, and the 457 noncarcinogenic chemicals were categorized as Class 0.

Using the individual chemical datasets mentioned above, we generated the following binary mixtures:

- Class 0: 459 individual Class 0 chemicals resulted in 105,111 binary mixtures.
- Class 1: 604 individual Class 1 chemicals resulted in 182,106 binary mixtures.
- Class 2: 555 individual Class 2 chemicals resulted in 153,735 binary mixtures.

For the multiclass classification model, we selected the following carcinogenic mixtures:

- 5000 randomly chosen mixtures (out of 105,111) from Class 0.
- 8000 randomly chosen mixtures (out of 182,106) from Class 1.
- 7000 randomly chosen mixtures (out of 153,735) from Class 2.

##### Preparation of Distinct Test Dataset: Separating Compounds into Training and Test Set

Multiclassification models were also developed by splitting compounds into training and test sets, ensuring that the compounds in the test set were not present in the training set.

(i) Class 0: The 459 compounds in Class 0 were divided into 367 for training and 92 for testing. All possible combinations resulted in 67,161 carcinogenic binary mixtures in the training set and 4186 mixtures in the test set.
(ii) Class 1: The 604 compounds in Class 1 were divided into 482 compounds for training and 122 compounds for testing. All possible combinations resulted in 115,921 carcinogenic binary mixtures in the training set and 7381 mixtures in the test set.
(iii) Class 2: The 555 compounds in Class 2 were divided into 443 for training and 112 for testing. All possible combinations resulted in 97,903 carcinogenic binary mixtures in the training set and 6216 in the test set.

The combined training set for the multiclass classification model consisted of 20,000 randomly selected binary mixtures, with 5000 from Class 0, 8000 from Class 1, and 7000 from Class 2. The test set consisted of 17,783 binary mixtures, with 4186 from Class 0, 7381 from Class 1, and 6216 from Class 2.

#### 2.1.5. Carcinogenicity Assessment of Virtual Mixtures Using Concentration Addition (CA) Regression Models: Assumptions and Methods

##### Assumption

We assumed that a mixture’s TD_50_ (median toxic dose) is the average of the TD_50_ values of its two component chemicals. This assumption is based on the concentration addition (CA) model, which assumes simple addition and a sum of toxic units (TUs) equal to 1 (see Section 2.2).

*CPDB Data*: A total of 157,080 carcinogenic binary mixtures were generated by considering all possible combinations of 561 single carcinogenic chemicals. The TD_50_ value of each individual chemical was utilized to calculate the concentration fraction, and the descriptors for the resulting mixtures were computed.

*Final Dataset*: For our regression model, we selected 20,000 carcinogenic mixtures randomly from the pool of 157,080 combinations.

Preparation of distinct test dataset: separating compounds into training and test set.

Regression models were developed by splitting the compounds into training and test sets and ensuring that the compounds in the test set were distinct from those in the training set.

*CPDB Data*: The 561 carcinogenic chemicals were divided into 449 for training and 112 for testing. All possible binary combinations resulted in 100,576 carcinogenic binary mixtures in the training set and 6216 mixtures in the test set.

The final training set for the regression model comprised 20,000 randomly selected carcinogenic mixtures from the pool of 100,576 combinations. The test set consisted of 6216 binary mixtures.

### 2.2. Regression Model

The concentration addition (CA) model [64,65] describes mixture toxicity as follows:

(1) $$EC_{50mix}=\frac{C_{M}}{\frac{C_{A}}{EC_{50A}}+\frac{C_{B}}{EC_{50B}}}=\frac{C_{M}}{TU_{A}+TU_{B}},$$

where *EC*_50*mix*_ represents the median effective concentration of the mixture, *C_A_*, *C_B_*, and *C_M_* denote the concentrations of components A, B, and the mixture, respectively, that are required to produce a median effect (50% effect) by the mixture, and *EC*_50*A*_ and *EC*_50*B*_ refer to the median effective concentration of components A and B when acting individually as single compounds. If the sum of toxic units (TU) at the median inhibition is equal to 1, it indicates a simple addition.

This study employed the concentration addition (CA) model with a simple addition approach to determine the mixture TD_50_ in the CPDB data. The TD_50_ of the mixture given by the CA model is as follows (from Equation (1)):

(2) $$TD_{50mix}=\frac{C_{M}}{\frac{C_{A}}{TD_{50A}\;}+\frac{C_{B}}{TD_{50B}}}=\frac{C_{M}}{TU}.$$

The sum of toxic units (TUs) of each component gives the joint TU of the mixture.

For simple addition,

(3) $$TU=\frac{C_{A}}{TD_{50A}}+\frac{C_{B}}{TD_{50B}}=1.$$

Considering the equitoxic ratio of the components A and B, the toxic unit of the two chemicals should be TU_A_:TU_B_ = 0.5:0.5. Thus,

TU=0.5+0.5,

$$C_{A}=0.5\;\times \;TD_{50A},\;C_{B}=0.5\;\times \;TD_{50B},$$

(4) $$C_{M}=C_{A\;}+C_{B}=0.5\;\times \;TD_{50A}+0.5\;\times \;TD_{50B}.$$

Hence, the TD_50_ of the mixture is the average of the TD_50_ of the component chemicals.

(5) $$TD_{50mix}=C_{A}+C_{B}=0.5\;\times \;TD_{50A}+0.5\;\times \;TD_{50B}.$$

With $TD_{50mix}$ available, the concentration of the component chemicals A and B can be calculated using the concentration fraction $p_{A}=\frac{C_{A}}{C_{M}}$ and $p_{B}=\frac{C_{B}}{C_{M}}$ as the scaling factor:

(6) $$C_{A}=p_{A}\times C_{M}\;=p_{A}\times TD_{50mix},$$

(7) $$C_{B}=p_{B}\times C_{M}\;=\;p_{B}\times TD_{50mix}.$$

### 2.3. Descriptor Calculation

#### Descriptor Calculation for Component Chemicals

SMILES is the chemical structure representation using ASCII strings. Descriptors were calculated using Mordred descriptor calculator [74] which calculates 1613 2D molecular descriptors from SMILES. We kept a final set of 653 descriptors with no missing values in any of the calculated descriptors.

### 2.4. Descriptor Calculation and Integration of Dose-Dependent Relationship for the Chemical Mixture: Mathematical Method and Process

#### 2.4.1. The Dose-Dependent Ratio of Different Chemical Components in a Mixture

All the collected data were converted to mol/L before calculating the log (1/EC_50_ or LC_50_, or ED_50_). Mole fractions of the component chemicals in a mixture were calculated from their median effective concentration when acting alone and their corresponding carcinogenicity or toxicity ratio in the mixture. Dose-dependent chemical mixture descriptor ‘D’ was calculated using three different mathematical formulas (sum, difference, and norm) as the basis as described previously [75].

*Sum*: The mixture descriptor ‘D’ was calculated as the sum of the molecular descriptors or fingerprints $d_{1}$, $d_{2}$…$d_{n}$ of the two or more components weighted by their respective mole fractions (dose-dependent) $x_{1}$, $x_{2}\;$…$x_{n}$ in the mixture:

(8) $$D=x_{1}d_{1}+x_{2}d_{2}+\cdots ..x_{n}d_{n}$$

*Difference*: The mixture descriptor D was calculated as the absolute difference between the molecular descriptors:

(9) $$D=\left|x_{1}d_{1}-x_{2}d_{2}-\cdots ..x_{n}d_{n}\right|$$

*Norm*: The mixture descriptor D was calculated as follows for the molecular descriptors:

(10) $$D=\sqrt{{\left(x_{1}d_{1}\right)}^{2}+{\left(x_{2}d_{2}\right)}^{2}}+\cdots .{\left(x_{n}d_{n}\right)}^{n}$$

In this study, we use and report only the sum method results. Mixture descriptors ‘D’ for the 653 descriptors were calculated using the sum method [73]. The mixture descriptor ‘D’ was calculated as the sum of the molecular descriptors $d_{1}$ and $d_{2}$ of the two component chemicals of the mixture, each scaled by their respective concentration fractions $x_{1}$ and $x_{2}$ in mg/L:

(11) $$D=x_{1}d_{1}+x_{2}d_{2}\;$$

#### 2.4.2. Structural Representation Descriptor Using SMILES of the Chemicals

The SMILES S for the mixture was generated by simple concatenation of the two SMILES strings S1 and S2 with a period (.) as the separator.

(12) S=S1.S2.

#### 2.4.3. SMILES Preprocessing

In our deep learning model, we utilized the ASCII strings of the SMILES representation of chemicals as an input feature. Python’s Tokenizer class was employed to encode the SMILES string into numerical form. We considered the 94 characters in the ASCII table, represented by decimal numbers 33 to 126, to ensure that no character in the SMILES string, regardless of its format, is missed. These 94 characters made the vocabulary of the possible characters in the SMILES. A dictionary, D = {‘!’:1, ‘”’:2, ‘#’:3, ‘$’:4, …, ‘C’:35, …, ‘~’:94}, was created that maps every character in the list of 94 ASCII characters to a unique index. A vector was created for the SMILES of each compound by converting each character in the SMILES string to its corresponding index in the dictionary. The resulting vector for the SMILES was padded with zeros or truncated so that it was of uniform length, L. We previously described in detail the SMILES preprocessing of chemicals [76,77,78].

### 2.5. Machine Learning Method

#### Hybrid Neural Network Method

We utilized the hybrid neural network (HNN) framework [76,77,78], which we developed for single chemical toxicity and carcinogen prediction. In contrast to the original model’s one-hot encoding of SMILES, here, we vectorized the SMILES using the method described in the SMILES preprocessing section. The HNN method, implemented using the Keras API in Python, comprises a convolutional neural network (CNN) for learning structural attributes (SMILES) and a feedforward neural network (FFNN) for learning from chemical descriptors. A CNN framework processes the complex matrix generated from the SMILES strings, while a multilayer perceptron (MLP) FFNN is employed to process and learn from the chemical descriptors and fingerprints. In this approach, the Keras embedding layer is utilized to convert each character’s index in the SMILES string into a dense vector of size 100. Consequently, the input 2D array of size K × L, where ‘K’ represents the number of SMILES and ‘L’ denotes the maximum length of SMILES, is transformed into a 3D array of size K × L × 100. The 3D array, consisting of one-hot encoded SMILES strings and image bytes, serves as the input for the CNN, while the chemical descriptors and fingerprints are provided as input to the FFNN. The output from the pooling layer of the CNN is merged with the final fully connected layer of the FFNN to perform the ultimate classification. For binary classification, the sigmoid activation function is applied. At the same time, for multiclassification, the fully connected layer utilizes the softmax activation function, generating ‘N’ probabilities, each corresponding to a specific class. For all methods, 30 simulations/iterations are executed, and the average is calculated for statistical metrics such as AUC, accuracy, selectivity, sensitivity, and precision.

### 2.6. Developing Binary and Multiclass Classification Models Using Alternative Machine Learning Methods

To compare and evaluate the predictions of the HNN method, we developed binary and multiclass classification models utilizing various machine learning techniques, including random forest (RF), bagged decision tree (bootstrap aggregating or bagging), and adaptive boosting (AdaBoost).

### 2.7. Developing Regression Models Using Alternative Machine Learning Methods

To compare and evaluate the predictions of the HNN method, we developed regression models using various machine learning methods, including random forest (RF), support vector regression (SVR), gradient boosting (GB), kernel ridge (KR), decision tree with AdaBoost (DT), and KNeighbors (KN). These models were implemented using the sklearn package in Python to generate the final consensus prediction of the mixture carcinogenicity. All these different machine learning methods were used for the comparative performances with our hybrid HNN method and to obtain consensus method predictions. The consensus predicted value was determined by calculating the average of the predicted values generated by the seven methods.

### 2.8. Model Evaluation

#### 2.8.1. Binary and Multiclass Classification Model

We employed a robust evaluation process to evaluate the mixture classification models’ performance. First, approximately 20% of the available data were randomly (until otherwise specified) allocated as the test set for each iteration to ensure an unbiased assessment. The evaluation process was repeated for 30 iterations, and the average results were used to gauge the model’s performance.

Several metrics were employed to assess the classification models. Accuracy, which measures the proportion of correctly classified instances, was utilized as a primary evaluation metric. Additionally, the area under the receiver operating characteristic curve (AUC) was calculated to assess the model’s ability to discriminate between different classes. Sensitivity, representing the true positive rate, and specificity, representing the true negative rate, were also considered to evaluate the model’s performance. These metrics provide insights into the model’s ability to identify positive and negative instances within the dataset correctly. Supplementary Equation (S1) provides further details on how these metrics were computed.

We utilized micro-averaging for multiclass classification to calculate the average value across all classes. This technique involved converting the data into binary classes and assigning equal weight to each observation, enabling a fair evaluation of the model’s performance across multiple classes. By considering the average performance across all classes, we obtained a comprehensive understanding of the model’s overall classification accuracy and performance.

#### 2.8.2. Regression Model

In the regression analysis, a similar evaluation process was conducted. In each of the 30 iterations, approximately 20% of the data were randomly set aside (until otherwise specified) as the test set to evaluate the model’s predictive performance. This random allocation ensured that the test set was representative of the entire dataset and reduced the potential for bias.

To assess the regression models, several metrics were employed. The mean square error (MSE), which quantifies the average squared difference between the predicted and actual values, provided a measure of the model’s overall prediction accuracy. The mean absolute error (MAE) was also utilized to evaluate the average magnitude of the errors made by the model. Additionally, the coefficient of determination (R^2^), a measure of the proportion of variance in the dependent variable explained by the model, was calculated to assess the goodness of fit. Supplementary Equation (S2) provides further details on how these metrics were computed.

By considering these metrics, we could comprehensively evaluate the regression models’ performance. The MSE and MAE metrics provided insights into the accuracy and precision of the predictions. In contrast, the R^2^ metric allowed us to assess the overall goodness of fit of the model to the data. Through this rigorous evaluation process, we obtained a thorough understanding of the classification and regression models’ performance in predicting the carcinogenic potential of chemical mixtures. These evaluation metrics enable us to assess the models’ accuracy, discrimination ability, and predictive capabilities, thereby ensuring the reliability and robustness of our findings.

All the results presented for the model evaluation are the average of 30 repeats. Approximately 20% of the data were separated randomly in each iteration as test sets, and the remaining data were taken as training sets subjected to fivefold cross-validation, except that the test sets were randomly selected in each iteration. For the fivefold cross-validation, we used 80:20 training to test set ratios, which are good numbers for the significant data size used in this study. Furthermore, the data were shuffled in each iteration before separating the training and the test set to ensure that the process did not end up with a dataset containing bias in both the training and the test set. Furthermore, the average performance metrics were calculated from the outcome of 30 simulations for the classification models. Training 80% of the data gave more room for better performance (compared to 10-fold cross-validation with 90% data in the training set) while predicting for external datasets using a model trained on 100% of the data.

Furthermore, to avoid bias and overfitting problems, after conducting initial tests on the predictive capability with randomly chosen training and test datasets, we evaluated HNN performance alongside various machine learning methods for the distinct training and test datasets. To achieve this, we simulated training and test datasets comprising distinct sets of chemicals. Here, our approach was to segregate compounds into the training and test sets, ensuring that each set consisted of unique compounds and combinations without any bias. This rigorous methodology was employed to avoid any potential biases or overlapping data between the training and test sets.

## 3. Results and Discussion

Here, we developed the hybrid neural network framework called HNN-MixCancer (referred to as HNN) to predict the carcinogenic potential of chemical mixtures in terms of carcinogenic or noncarcinogenic, categorical carcinogenicity, and carcinogenic potency. To simulate the chemical mixtures, a mathematical framework was combined with the HNN framework. To assess the prediction performance of the HNN models, we constructed different classification models such as binary model (carcinogenic or noncarcinogenic), multiclass classification model (categorical carcinogenicity) and regression model (carcinogenic potency). To build these models, we utilized a combination of experimental data from the literature that provided information on chemical mixtures. However, given the limited availability of experimental mixture data, we also generated assumption-based virtual chemical mixtures to enrich the training dataset. This approach allowed us to compensate for the lack of empirical data and improve the robustness of our models. To compare and improve the prediction performance of the HNN method, we also developed classification models based on several other methods, including random forest (RF), Bootstrap aggregating (bagging), adaptive boosting (AdaBoost), support vector regressor (SVR), gradient boosting (GB), kernel ridge (KR), decision tree with AdaBoost (DT), and KNeighbors (KN). These alternative methods were employed to develop binary, multiclass, and regression models, ensuring a comprehensive evaluation of the prediction performance. We also utilized these methods to generate an ensemble model for consensus prediction.

The binary classification models were designed to determine whether a chemical mixture is carcinogenic or noncarcinogenic, providing a clear classification of its potential risk. On the other hand, the multiclass classification models aimed to categorize chemical mixtures into different classes on the basis of their degree of carcinogenicity. These models facilitated a more nuanced understanding of the mixture’s potential effects, allowing for a finer classification of their carcinogenic potential. When dose information was absent, both the binary and the multiclass models relied on equal concentrations of the component chemicals to calculate the concentration fraction and derive a weighted mixture descriptor.

Regression models, on the other hand, were employed to predict the effective concentration at which a chemical mixture becomes carcinogenic. These models assumed a simple addition of the concentration addition (CA) model, incorporating an equitoxic ratio of the component chemicals to calculate the mixture’s carcinogenicity. The regression models leveraged this information to estimate the specific concentration at which the mixture exhibits carcinogenic effects. To obtain the mixture descriptor, the weight was calculated using the TD_50_ (median toxic dose) of the single carcinogens, and the mixture TD_50_ was determined by averaging the TD_50_ values of the component chemicals. This approach allowed for the quantification and characterization of the carcinogenic potential of the chemical mixtures.

By employing these diverse models within the HNN framework, we aimed to provide accurate predictions of the dose-dependent carcinogenic potential of chemical mixtures. Through the binary and multiclass models, we obtained valuable insights into the classification of mixtures based on their carcinogenicity. In contrast, the regression models offered quantitative estimations of the effective concentrations at which carcinogenic effects occur. This comprehensive modeling approach allowed us to capture the complex relationships between mixture components, concentrations, and their carcinogenic potential, ultimately enhancing our understanding of the risks associated with chemical mixtures. The detailed results and their discussions are presented below.

### 3.1. Carcinogenicity Prediction through Binary Classification Using Literature and DCDB Data

**Figure 1:**
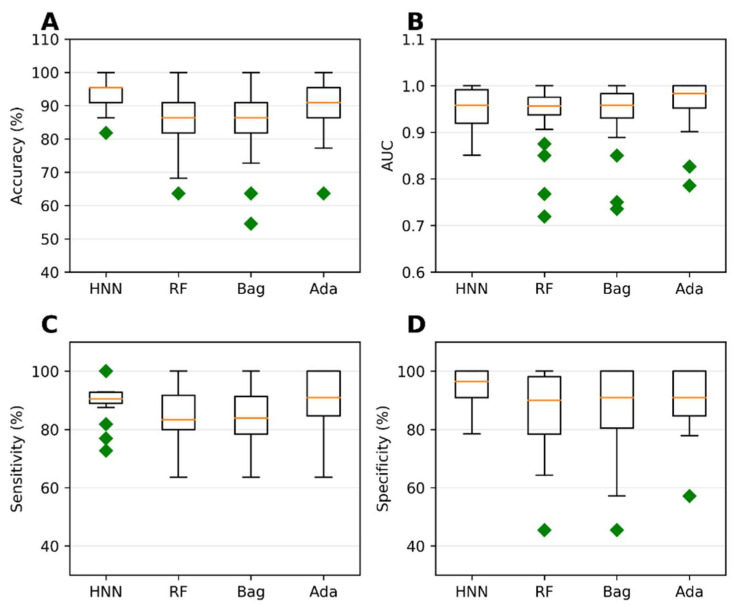
The HNN, RF, bagging, and AdaBoost models were utilized for binary classification and evaluated using the following statistical metrics: (**A**) accuracy, (**B**) AUC, (**C**) sensitivity, and (**D**) specificity. The models were applied to mixtures sourced from both the literature and the DCDB database. Green diamond represents outliers.

Binary classification models were developed using the HNN, RF, bagging, and AdaBoost methods to predict carcinogenicity. The models were trained on a dataset consisting of 54 carcinogenic and 25 noncarcinogenic combinations from the literature, along with 30 drug combinations from the DCDB database classified as noncarcinogens. Among the models, the HNN demonstrated the highest accuracy of 92.72%, sensitivity of 90.85%, and specificity of 94.82% (Figure 1A,C,D). Additionally, the HNN and AdaBoost achieved the highest AUC of approximately 0.96 compared to other methods (Figure 1B). In summary, the HNN outperformed other machine learning methods in terms of accuracy and achieved the highest AUC for carcinogenicity prediction.

### 3.2. Predicting Carcinogenicity through Binary Classification Using Assumption-Based Virtual Binary Mixtures

During our comprehensive survey of various data sources, it became apparent that there was a lack of experimental data in the literature that could be used to train the binary mixture models required for our research on carcinogenicity prediction. In light of this limitation, we developed a strategy to address the issue by creating a virtual library of binary mixtures. This involved incorporating several assumptions, which were thoroughly explained in Section 2. By employing this approach, we successfully expanded the training dataset by including both carcinogenic and noncarcinogenic mixtures. Subsequently, we utilized this augmented dataset to construct accurate and reliable machine learning models. A noteworthy advantage of utilizing assumption-based binary mixtures is the ability to encompass a diverse range of structural variations within our dataset. This inclusion significantly improves the representativeness and robustness of our models, enabling them to capture a broader spectrum of real-world scenarios.

#### 3.2.1. Carcinogenicity Prediction of Chemical Mixtures Using Randomly Selected Training and Test Sets

To assess the predictive capabilities of the HNN (hybrid neural network) alongside other machine learning methods, we commenced the evaluation process by simulating randomly selected mixtures for both the training and the test datasets. This approach allowed us to assess the performance and generalization ability of the models when challenged with new and unknown or unfamiliar carcinogenic data. By employing this rigorous testing methodology, we aimed to obtain reliable and unbiased predictions of carcinogenicity for chemical mixtures.

##### Carcinogenicity Prediction Using Binary Classification

**Figure 2:**
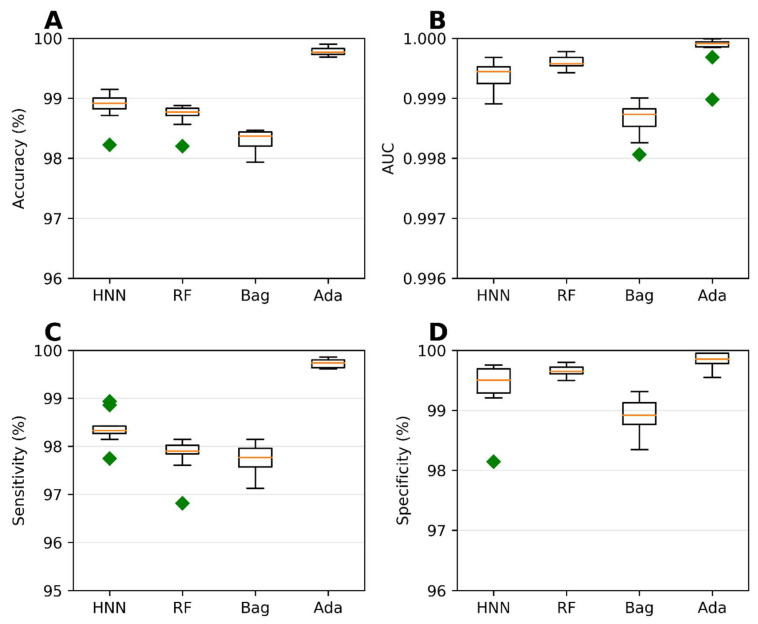
Statistical metrics of binary classification models: the HNN, RF, Bagging, and AdaBoost models were assessed using the following statistical metrics for assumption-based virtual binary mixtures generated from the MEG and CPDB databases of carcinogens and noncarcinogens: (**A**) accuracy, (**B**) AUC, (**C**) sensitivity, and (**D**) specificity. Green diamond represents outliers.

To assess the carcinogenic potential of virtual binary mixtures using a binary classification model, we formed carcinogenic mixtures by pairing carcinogens with other carcinogens. Conversely, noncarcinogenic mixtures were created by pairing noncarcinogens with other noncarcinogens. To compile our dataset of binary mixtures, we leveraged data from two reliable sources: the MEG and CPDB. Subsequently, binary classification models were developed using the HNN, RF, bagging, and AdaBoost methods, considering 20,568 binary mixtures. In each simulation, a randomly selected 20% of the mixtures were reserved as a test set. The AdaBoost and HNN methods exhibited exceptional predictive performance among the binary classification models. Their statistical metrics, including accuracy, AUC, sensitivity, and specificity, surpassed 99% (Figure 2). These findings highlight the superior capabilities of the AdaBoost and HNN in accurately predicting carcinogenicity within the context of binary mixtures.

##### Validation of the Binary Classification Models

**Figure 3:**
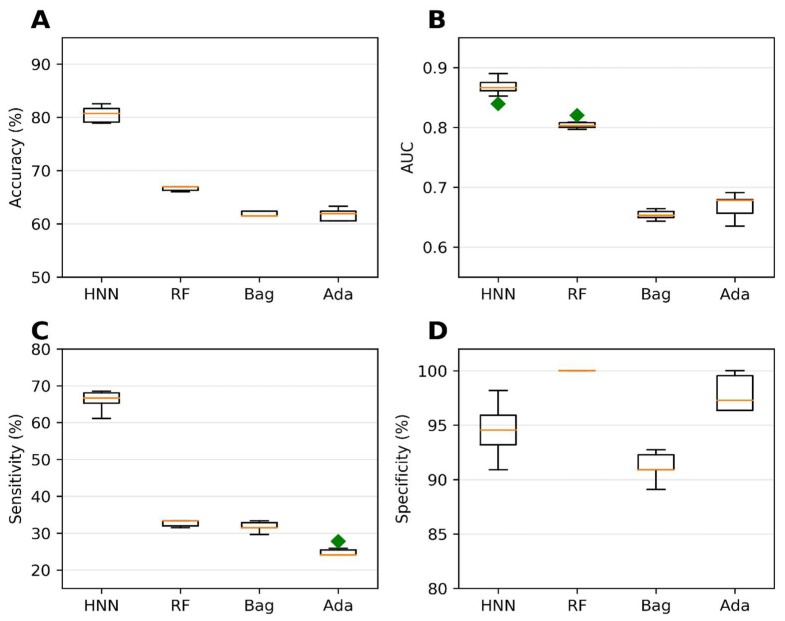
Statistical metrics of the binary classification models (HNN, RF, bagging, and AdaBoost) evaluated on the assumption-based virtual binary mixtures training dataset generated from the MEG and CPDB databases of carcinogens and noncarcinogens: (**A**) accuracy, (**B**) AUC, (**C**) sensitivity, and (**D**) specificity. The external validation test set was developed using data collected from the literature and the DCDB database. Green diamond represents outliers.

Validation of the binary classification models was the next step in our study. The purpose was to conduct external validation to assess the prediction capability, reproducibility, and generalizability of the HNN to new and diverse datasets. The evaluation of the predictive ability of the binary classification models was performed using an external validation set as the test set. The external dataset used consisted of 79 mixture data obtained from the literature and 30 drug combinations sourced from the DCDB. We utilized a training set comprising 20,568 virtual binary mixtures to train the models. These mixtures were developed on the basis of binary mixture data from MEG and CPDB. The use of such a large training set enabled comprehensive coverage of various chemical combinations, contributing to the robustness of the models. Among the methods tested, the HNN demonstrated the highest accuracy of 80.55% during the external validation (Figure 3A). This accuracy represents the HNN’s ability to classify carcinogenic and noncarcinogenic mixtures correctly. The HNN-generated model also achieved an AUC of 0.86, indicating a robust discriminatory power in distinguishing between the two classes (Figure 3B). Moreover, the sensitivity of the HNN model was determined to be 66.29%, indicating its capacity to identify carcinogenic mixtures (Figure 3C) accurately. In contrast, the RF (random forest) model exhibited the best average specificity of 99.5% during the external validation (Figure 3D). This signifies the RF model’s ability to correctly classify noncarcinogenic mixtures with high precision, minimizing false-positive predictions.

The exceptional predictive performance observed during the external validation demonstrates the HNN method’s ability to predict the carcinogenicity of chemical mixtures. The high accuracy, AUC, and sensitivity obtained by the HNN provide strong evidence of its efficacy in identifying potential carcinogens accurately. Furthermore, the RF model’s excellent specificity further highlights its capability to distinguish noncarcinogenic mixtures reliably. In summary, the external validation process confirmed the binary classification models’ predictive capability, reproducibility, and generalizability. The HNN model exhibited excellent accuracy, AUC, and sensitivity in predicting the carcinogenicity of chemical mixtures, while the RF model excelled in specificity. These findings instill confidence in the HNN method’s flexibility to predict the carcinogenic potential of various unknown chemical mixtures.

##### Carcinogenicity Prediction Using Multiclass Classification

To enable the prediction of categorical carcinogenicity of chemical mixtures, we next proceeded to develop multiclass classification models. For this purpose, categorical data were collected from multiple sources, including MEG, NTP, IARC, and JSOH. Detailed information regarding the data sources, collection, training, and test set preparation can be found in Section 2. The chemicals included in the multiclass models were initially categorized into three classes: Class 0, representing noncarcinogens; Class 1, indicating possibly carcinogens and not classifiable chemicals; Class 2, representing carcinogens and probably carcinogens. This classification scheme allowed for a comprehensive assessment of the carcinogenic potential of chemical mixtures.

To construct the training sets, compounds within each class were paired together, creating mixtures belonging to the same class. The training set comprised 20,000 binary mixtures, which served as a foundation for training the multiclass classification models. Additionally, 20% of the mixtures were randomly selected for each simulation to form the test set, ensuring an unbiased evaluation of the models’ performance. The multiclass classification models were developed using various methods, including HNN, RF, bagging, and AdaBoost. These methods encompassed a range of algorithms and techniques, each with its own strengths and characteristics.

**Figure 4:**
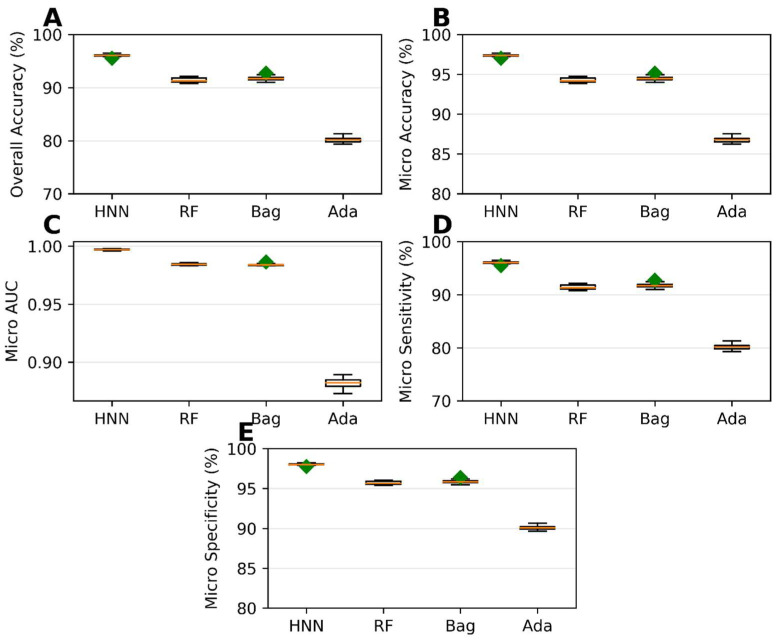
Statistical metrics of multiclass classification models: (**A**) overall accuracy, (**B**) micro-accuracy, (**C**) micro-AUC, (**D**) micro-sensitivity, and (**E**) micro-specificity for assumption-based virtual binary mixtures training dataset generated from MEG, NTP, IARC, and JSOH. Green diamond represents outliers.

The HNN method exhibited the best predictive performance among the multiclass classification models. It achieved an overall accuracy of 96.03%, indicating its ability to correctly classify chemical mixtures into their respective classes (Figure 4A). The micro-accuracy was 97.35%, further highlighting the HNN method’s capability to predict individual classes (Figure 4B) accurately. Additionally, the HNN model achieved a micro-AUC of 0.99, indicating a robust discriminatory power in distinguishing between the different classes (Figure 4C). The micro-sensitivity and micro-specificity of the HNN model were found to be 96.03% and 98.01%, respectively (Figure 4D,E), demonstrating its ability to identify both positive and negative cases correctly. In contrast, the AdaBoost method showed the lowest predictability among the multiclass classification models (Figure 4). While the specific performance metrics for AdaBoost are not mentioned, it was observed that its predictive performance fell behind the other methods in terms of overall accuracy, AUC, sensitivity, and specificity. In summary, the development of multiclass classification models enabled the prediction of the categorical carcinogenicity of chemical mixtures. The HNN method exhibited superior predictive performance, achieving high accuracy, AUC, sensitivity, and specificity. This indicates its efficacy in accurately classifying chemical mixtures into their respective classes. On the other hand, AdaBoost showed the lowest predictability among the methods for multiclassification.

##### Carcinogenicity Prediction Using Regression

**Figure 5:**
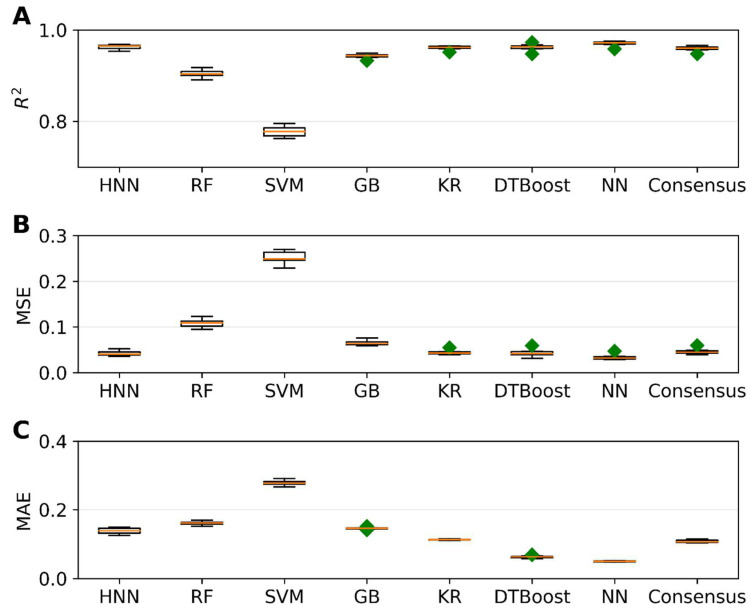
The statistical metrics of various regression models, namely HNN, RF, SVR, GB, KR, DTBoost, NN, and consensus, in relation to assumption-based mixtures: (**A**) coefficient of determination (R^2^), (**B**) mean squared error (MSE), and (**C**) mean absolute error (MAE). The HNN, RF, SVR, GB, KR, DTBoost, and NN methods were utilized to generate a consensus prediction. Green diamond represents outliers.

Next, we aimed to predict carcinogenic potency by utilizing various regression models. To accomplish this, we employed the carcinogenic potency TD_50_ data sourced from the CPDB, as outlined in Section 2. The regression models were developed using different methods, including HNN, RF, SVR, GB, KR, DT, and KN. To evaluate the performance of these regression models, we utilized three metrics: coefficient of determination (r^2^), mean-square error (MSE), and mean absolute error (MAE). The results of our analysis are presented in Figure 5. We randomly selected 20% of the 20,000 binary mixtures for each simulation as the test set, while the remaining 80% comprised the training set. Figure 5A demonstrates that, except for the SVM method, all other methods achieved an r^2^ value greater than 0.94. Notably, the HNN and NN methods exhibited the highest r^2^, exceeding 0.96. Additionally, these methods yielded the lowest MSE of 0.03 (Figure 5B) and MAE of 0.05 (Figure 5C), respectively, showcasing their superior performance. In summary, by comparing various methods, we found that the HNN and NN approaches yielded the most accurate predictions, as indicated by their high r^2^ values and low MSE and MAE scores. These findings highlight the potential of the HNN method in accurately estimating carcinogenic potency.

#### 3.2.2. Predicting Carcinogenicity of Chemical Mixtures with Distinct Training and Test Set Data

Next, after conducting initial tests on the predictive capability of the hybrid neural network (HNN), we evaluated its performance alongside various machine learning methods for the distinct training and test datasets. To achieve this, we simulated training and test datasets comprising distinct chemical compounds. Our approach involved developing classification models by segregating compounds into the training and test sets, ensuring that each set consisted of unique compounds and combinations. By doing so, we ensured that none of the compounds present in the test set were included in the training set. This rigorous methodology was employed to avoid any potential biases or overlapping data between the training and test sets. It is important to note that we meticulously avoided selecting randomly chosen chemicals from the training set for each simulation to form the test set. This deliberate selection process ensured that the test set remained entirely independent, enabling a robust evaluation of the model’s predictive capabilities. By adopting this comprehensive strategy, we aimed to provide a fair and unbiased assessment of the HNN’s performance compared to other machine learning techniques in the context of chemical compound classification.

##### Carcinogenicity Prediction Using Binary Classification

**Figure 6:**
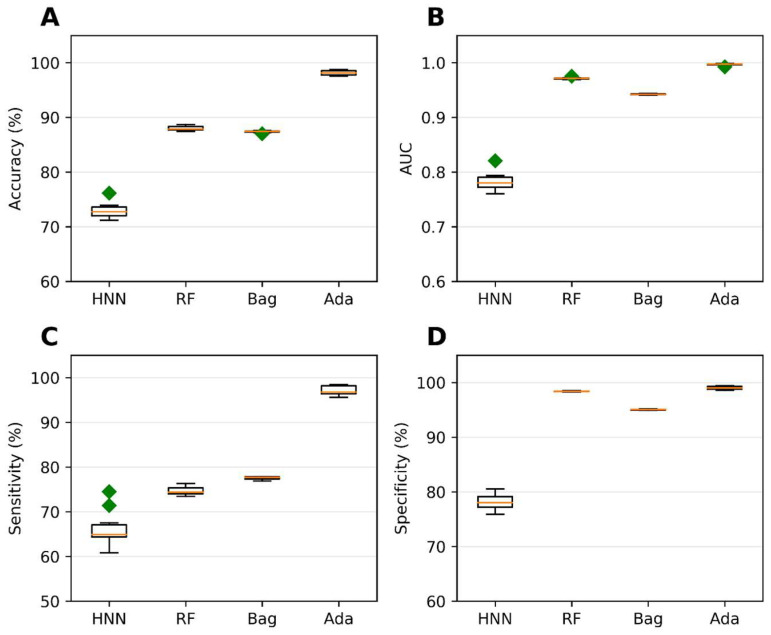
Statistical metrics of binary classification models for assumption-based virtual binary mixtures, including HNN, RF, bagging, and AdaBoost: (**A**) accuracy, (**B**) AUC, (**C**) sensitivity, and (**D**) specificity. Datasets were generated from MEG and CPDB databases of carcinogens and noncarcinogens. Green diamond represents outliers.

The single carcinogens obtained from the MEG and CPDB datasets were divided into training and test sets to predict the carcinogenicity of chemical mixtures. The binary mixtures were then formed by pairing one carcinogen with another carcinogen, while the binary noncarcinogen mixtures were created by pairing noncarcinogens. We employed the HNN, RF, bagging, and AdaBoost methods to develop binary classification models using the training set, which consisted of 20,089 binary mixtures. Subsequently, we evaluated the performance of these models on the test set, which comprised 11,851 binary mixtures. Among the different methods, the AdaBoost models demonstrated the highest predictive performance. They achieved an accuracy of 98.16%, an area under the receiver operating characteristic curve (AUC) of 0.996, a sensitivity of 97.07%, and a specificity of 99%. These results, as shown in Figure 6, highlight the effectiveness of the AdaBoost approach in accurately classifying the carcinogenic potential of binary mixtures for the case of distinct datasets. Conversely, the performance of the HNN model was less accurate in this context, which needs improvement in the distinct case, as discussed in Section 4. It is worth noting that separating the training and test sets yielded improved predictive accuracy compared to the models developed without separating these sets (as shown in Figure 2 and Figure 6, Supplementary Table S3). This suggests that the separation of datasets can contribute to better model performance, particularly in the case of binary classification for carcinogenicity prediction. In summary, we successfully predicted the carcinogenic potential of chemical mixtures by utilizing binary classification models. Moreover, separating the training and test sets further enhanced the accuracy of the models.

##### Carcinogenicity Prediction Using Multiclass Classification

**Figure 7:**
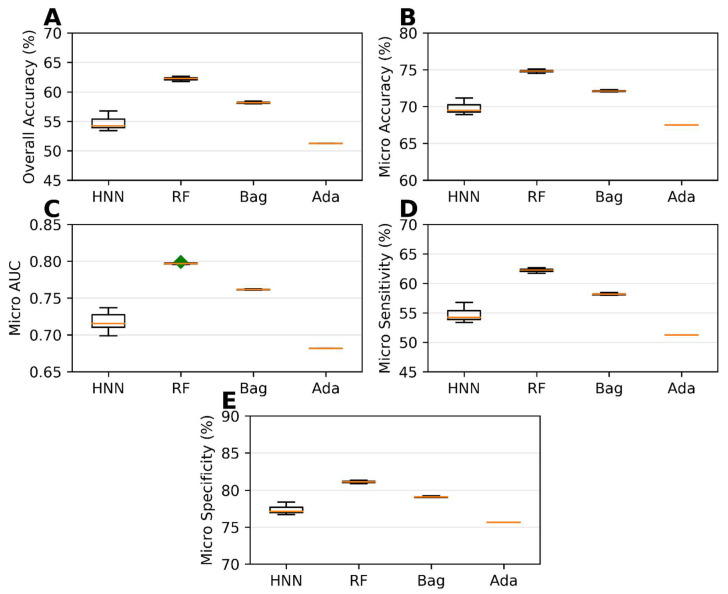
Statistical metrics of multiclass classification models (HNN, RF, bagging, and AdaBoost) for assumption-based virtual mixtures with separated training and test datasets: (**A**) overall accuracy, (**B**) micro-accuracy, (**C**) micro-AUC, (**D**) micro-sensitivity, and (**E**) micro-specificity. Green diamond represents outliers.

Carcinogenicity prediction using multiclass classification involved the classification of single carcinogens obtained from MEG, NTP, IARC, and JSOH into different class categories: Class 0, Class 1, Class 2, and Class 3. This classification process was described in detail in Section 2. The single carcinogens were then divided into training and test sets before creating mixtures. The training set comprised 20,000 binary mixtures, while the test set consisted of 17,783 binary mixtures. Different multiclass classification models, namely, HNN, RF, bagging, and AdaBoost, were developed using the training set. These models were then evaluated for their predictive performance using the test set. Among the models, the RF models demonstrated the highest accuracy, micro-accuracy, micro-AUC, micro-sensitivity, and micro-specificity. Specifically, the RF models achieved an overall accuracy of 62.22%, a micro-accuracy of 74.81%, a micro-AUC of 0.79, a micro-sensitivity of 62.22%, and a micro-specificity of 81.11% (Figure 7). The HNN method performance was close to optimal. On the other hand, the AdaBoost method yielded the lowest performance metrics in this case of multiclassification, as shown in Figure 7. However, it is noteworthy that, when the training and test sets were separated, the highest overall accuracy obtained significantly decreased compared to the models built without separating these sets. This information is illustrated in Figure 4 and Figure 7, as well as in Supplementary Table S4).

##### Carcinogenicity Prediction Using Regression Model

**Figure 8:**
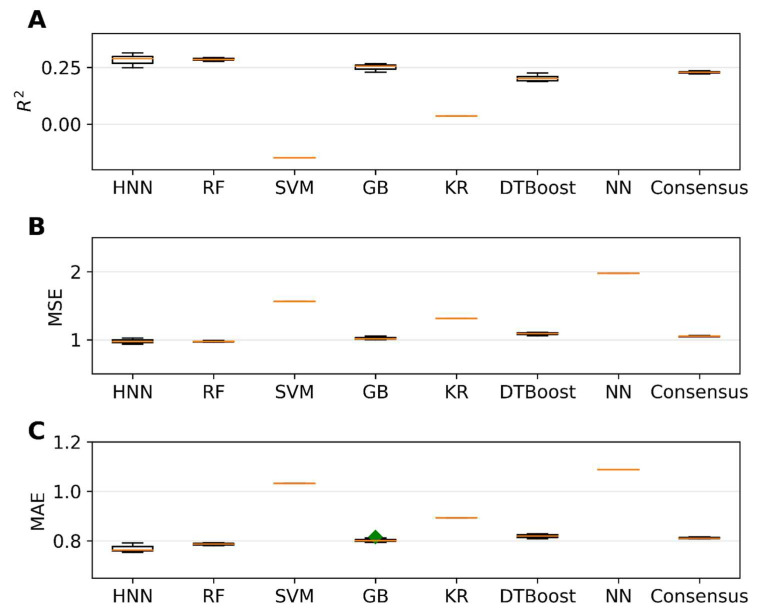
The statistical metrics of the HNN, RF, SVR, GB, KR, DTBoost, NN, and consensus regression models: (**A**) coefficient of determination (R^2^), (**B**) mean square error (MSE), and (**C**) mean absolute error (MAE) for the assumption-based mixtures with a separated dataset. Green diamond represents outliers.

The single carcinogens from the CPDB dataset were separated into training and test sets before forming their mixtures. The mixtures were formed with 20,000 binary mixtures in the training set, and the predictive performance of the models was tested for the 6216 binary mixtures in the test set. The HNN, RF, SVR, GB, KR, DT, and KN-based regression models were developed for the 20,000 binary mixtures in the training set, and the predictive performance was tested on the 6216 binary mixtures in the test set. The HNN method gave the highest r^2^ of 0.38, along with the lowest MSE of 0.97 and lowest MAE of 0.76 (Figure 8). In the regression model, the predictive performance of the models reduced drastically when the single chemicals were separated into training and test sets as compared to the regression models built without separating the training and sets (Figure 5 and Figure 8, Supplementary Table S5).

In summary, several trends emerge when comparing the performance of machine learning models between distinct training and test datasets (where the training and test data were completely separated) and nonseparated datasets (where the test data were randomly selected from within the training set). The predictive performance of the models showed the following patterns: a slight decrease for binary classification, a significant decrease for multiclass classification, and a drastic decrease for regression models.

We investigate these trends below and explore the reasons behind them. In binary classification, the machine learning task involves predicting the carcinogenicity of a chemical, which means there are only two possible outcomes: carcinogen or noncarcinogen. When the training and test data are separated, the models tend to exhibit a slight decrease in performance. This can be attributed to the fact that the models are trained on a specific distribution of data and may struggle to generalize well to new instances in the test set.

Moving on to multiclass classification, the scenario becomes more complex as there are three possible outcomes: Class 0, Class 1, and Class 2. In this case, when the training and test data are separated, the models experience a significant decrease in performance. The increasing number of classes to be represented poses a challenge for the models, as they need to learn more intricate decision boundaries and capture the distinctions among different classes. With the separation of training and test data, the models may have encountered only some classes during training, leading to reduced accuracy in predicting them during testing.

Regression models, which deal with continuous values and can yield an indefinite number of outcomes, exhibit a drastic decrease in performance when the training and test data are separated. Regression tasks involve predicting numerical values, such as predicting the toxicity level of a chemical. When a chemical present in the test set also appears in the training set, the models can make predictions with a higher degree of certainty, as they have learned patterns and correlations specific to that chemical. However, the uncertainty increases when a chemical in the test set is distinct from the training set (meaning it does not appear in the training set). The growing number of potential outcomes, combined with the lack of familiarity with the distinct chemical, makes it challenging for the models to predict its toxicity level accurately.

Taken together, when training and test data are separated, machine learning models experience varying degrees of decreased predictive performance. Binary classification models show a slight decrease, multiclass classification models experience a significant decrease, and regression models exhibit a drastic decrease. These trends can be attributed to factors such as the increasing number of classes or outcomes to be represented and the uncertainty that arises when encountering distinct instances in the test set that were not encountered during training. Understanding these trends can help guide designing and evaluating machine learning models in different scenarios, ensuring more accurate and reliable predictions.

## 4. Limitations

The performance of the hybrid neural network (HNN) model in predicting the carcinogenicity of binary mixtures was found to be reduced when using different virtual mixtures in the training and testing datasets, i.e., distinct datasets. This highlights the need for enhancements in several aspects of the model, including its architecture, hyperparameters, training process, and data representation. By addressing these limitations, the HNN model can be better tailored to the specific task of classifying the carcinogenic potential of binary mixtures. Despite outperforming other regression models and demonstrating optimal performance in multiclassification tasks, the HNN method still needs to achieve statistically superior results. The moderate accuracy observed in the binary classification of virtual mixtures, along with the limited explanatory power exhibited by the regression model on the distinct virtual mixture test dataset, can be attributed to the inherent complexity and variability involved in accurately predicting carcinogenicity. Carcinogenicity is influenced by various factors, such as molecular properties, chemical interactions, and biological mechanisms, all of which contribute to the challenges faced by regression-based approaches in capturing the underlying relationships accurately.

Furthermore, when individual chemicals are separated into distinct training and test sets, it can potentially undermine the HNN model’s effectiveness in learning and generalizing from the data. This separation can lead to the loss of crucial information and disrupt the underlying patterns within the neural network. Moreover, it may overlook important contextual information related to the combined behavior of chemicals within mixtures, impeding the model’s recognition of these chemicals’ interconnectedness and collective influence on carcinogenic potential. Consequently, the model’s capacity to capture the complex interplay of molecular properties, chemical interactions, and biological mechanisms contributing to carcinogenicity might be compromised. To overcome these challenges, strategies must be developed to acknowledge the holistic nature of binary mixtures. Instead of isolating individual chemicals, an approach that integrates the chemical components within the training and test sets can provide a more comprehensive representation of the underlying patterns. By preserving the combined behavior of mixtures, the model can more accurately capture the intricate relationships and interactions among the chemicals. This comprehensive approach enhances the model’s predictive performance by effectively learning the complex interdependencies and synergistic effects within binary mixtures. Significant improvements can be achieved in the model’s predictive performance by addressing the limitations posed by the separation of individual chemicals and implementing methodologies that preserve the holistic nature of binary mixtures. This comprehensive approach strengthens the model’s ability to accurately predict the carcinogenicity of binary mixtures by capturing the intricate relationships and interactions among the chemicals. Additionally, including additional cases beyond the studied Case 1 and Case 2 in the binary classification models is expected to enhance the predictive capabilities of the HNN model. These additional cases, coupled with a comprehensive approach of separating the training and test datasets, will be incorporated into the next version of the model as part of ongoing improvements.

Additionally, to further enhance the overall predictive performance of the HNN, an extensive array of optimization techniques will be applied. This will involve fine-tuning the model’s architecture and hyperparameters tailored explicitly to this dataset to achieve optimal performance. Potential improvements can be achieved by exploring adjustments to the number of hidden layers, neurons per layer, activation functions, and other architectural choices. Furthermore, refining the training process by optimizing the learning rate or incorporating regularization techniques may also lead to enhanced performance. To ensure the quality and representativeness of the training data, it is crucial to construct a diverse and well-balanced training set that adequately captures the essential characteristics of binary mixtures. To further improve the accuracy of the HNN, data augmentation techniques can be employed to introduce additional variations and increase the diversity of the training samples. Additionally, including additional relevant features holds the potential to enhance the accuracy of the HNN. For instance, incorporating molecular descriptors or biological data as additional features can provide comprehensive insights into the task of carcinogenicity prediction and significantly improve the model’s performance.

In this study, we considered equal doses or different concentrations of the component chemicals on the basis of the available literature. It has been well documented that chronic exposure to mixtures of chemicals, which are individually noncarcinogenic at very low doses, can lead to carcinogenesis through synergistic interactions involving cancer-related mechanisms [79]. Thus, it would be advantageous to consider the individual doses of chemicals when predicting the carcinogenicity of binary mixtures and estimating the median effective dose of the component chemicals in regression models. This approach allows for a more accurate and realistic evaluation of mixture carcinogenicity. To achieve a more comprehensive understanding of mixture carcinogenesis, it is essential to model synergistic interactions that are specific to human cells [80]. Animal models may not accurately reflect human responses; therefore, incorporating information specific to human cells in future predictions will contribute to a more reliable assessment of mixture carcinogenicity. Furthermore, this study serves as a proof of concept in developing classification-focused mixture models that combine elements of both whole mixture- and component-based approaches. The ongoing development of the HNN, the updated version of the model, will incorporate all the aforementioned enhancements. Future iterations of the model will include complete dose–response data, mode of action, combined action (independent, synergistic, or additive), and biological response data. These additions will enable a more robust evaluation of carcinogenicity in mixtures, providing a more comprehensive understanding of the complex interactions involved.

## 5. Conclusions

Safeguarding human health and safety from hazardous chemical exposure remains the primary objective of public health management. The mounting body of evidence supports the association between hazardous chemical exposure and cancer incidence. Therefore, it is imperative for environmental protection and health agencies worldwide to focus on characterizing the carcinogenicity of chemical mixtures. This study introduces HNN_MixCancer_, a new machine-learning framework that employs a hybrid neural network (HNN) to estimate the potential carcinogenicity of chemical mixtures at varying doses. Our framework combines cutting-edge machine-learning techniques with a mathematical model to simulate the behavior of mixtures. The results obtained demonstrate the exceptional predictive power of HNN_MixCancer_ in prioritizing carcinogenic chemical mixtures, even in cases where experimental data on mixtures are limited. In binary classification, the HNN outperforms other prominent machine learning methods such as random forest, bootstrap aggregating, adaptive boosting, support vector regressor, gradient boosting, kernel ridge, decision tree with AdaBoost, and KNeighbors, achieving an impressive accuracy of 93% and an AUC of 0.96. External validation on a known mixture test set further confirms its effectiveness with an accuracy of 81%. In multiclass classification, the HNN attains an overall accuracy of 96%, surpassing methods such as RF, bagging, and AdaBoost. The regression models based on HNN, RF, SVR, GB, KR, DT with AdaBoost, and KN exhibit high R^2^ values (ranging from 0.90 to 0.97), indicating their efficacy in predicting the concentration at which a chemical mixture becomes carcinogenic.

Furthermore, we employed assumption-based mixtures to enrich the carcinogenicity dataset, and HNN demonstrated a high level of reliability in predicting the carcinogenic potential of virtual binary mixtures with an accuracy of 81%. In multiclass classification, the HNN achieved the highest overall accuracy of 96% and micro-accuracy of 97%, outperforming other methods. However, it should be noted that when single chemicals were separated into training and test sets, predictive performance decreased for all methods in binary, multiclass, and regression classifications due to the increased number of possible outcomes.

Overall, our HNN method retained the highest predictive power for prioritizing carcinogenic chemical mixtures and performed exceptionally well in external validation. The promising validation results obtained from in vitro and in vivo PDX models further validate the predictive capability of HNN, and such additional findings will be detailed in our forthcoming manuscripts. To the best of our knowledge, this study is the first to present a method for predicting the carcinogenic potential of chemical mixtures using various classification models and machine learning techniques. It encompasses binary classification, categorical carcinogenicity classification, and the estimation of carcinogenic potency. Ongoing optimization and refinement of HNN_MixCancer_ will aim to address the limitations discussed. The method can be readily applied to evaluate the carcinogenic potential of diverse chemical mixtures including chemical type such as PFAS mixtures and co-occurring chemicals while considering the doses of component chemicals. Moreover, an enhanced version of our HNN method holds significant value for regulatory purposes. Although the mixture models are currently in the proof-of-concept stage, we intend to make future versions and predictions of chemical mixture carcinogenicity publicly accessible through a user-friendly online web server.

## Data Availability

Supporting Data are available in the Supplementary Materials.

## Supplementary Materials

The following supporting information can be downloaded at: https://www.mdpi.com/article/10.3390/toxics11070605/s1, Table S1: Carcinogenic binary chemical combinations collected from publications; Table S2: non-carcinogenic binary drug and chemical combinations collected from publications; Table S3: Change in % accuracy for distinct training and test sets of virtual mixtures for the binary classification; Table S4: Change in % accuracy for distinct training and test sets of virtual mixtures for the multiclass classification; Table S5: Change in coefficient of determination (R2) for distinct training and test sets of virtual mixtures for the regression classification. Equation (S1): Equations to calculate the evaluation metrics for binary & multiclass classification; Equation (S2): Equations to calculate the evaluation metrics for regression models; Other Supplementary Materials: Different cases assumption scenarios for creating a virtual binary mixture.

## References

[1] Marone P.A., Hall W.C., Hayes A.W. (2014). Reassessing the Two-Year Rodent Carcinogenicity Bioassay: A Review of the Applicability to Human Risk and Current Perspectives. Regul. Toxicol. Pharmacol. RTP.

[2] Adams B.L., Whitten R.O. (2018). Hepatocellular Carcinomas Are Promoted by Tocopheryl Acetate but Eliminated by Tocopheryl Succinate. J. Nutr. Intermed. Metab..

[3] Bigsby R.M. (2002). Synergistic Tumor Promoter Effects of Estrone and Progesterone in Methylnitrosourea-Induced Rat Mammary Cancer. Cancer Lett..

[4] Carlson D.B., Williams D.E., Spitsbergen J.M., Ross P.F., Bacon C.W., Meredith F.I., Riley R.T. (2001). Fumonisin B1 Promotes Aflatoxin B1 and N-Methyl-N’-Nitro-Nitrosoguanidine-Initiated Liver Tumors in Rainbow Trout. Toxicol. Appl. Pharmacol..

[5] Gábelová A., Poláková V., Prochazka G., Kretová M., Poloncová K., Regendová E., Luciaková K., Segerbäck D. (2013). Sustained Induction of Cytochrome P4501A1 in Human Hepatoma Cells by Co-Exposure to Benzo[a]Pyrene and 7H-Dibenzo[c,g]Carbazole Underlies the Synergistic Effects on DNA Adduct Formation. Toxicol. Appl. Pharmacol..

[6] Staal Y.C.M., Hebels D.G.A.J., van Herwijnen M.H.M., Gottschalk R.W.H., van Schooten F.J., van Delft J.H.M. (2007). Binary PAH Mixtures Cause Additive or Antagonistic Effects on Gene Expression but Synergistic Effects on DNA Adduct Formation. Carcinogenesis.

[7] Arjona-Sánchez A., Ruiz-Rabelo J., Perea M.D., Vázquez R., Cruz A., Muñoz M.d.C., Túnez I., Muntané J., Padillo F.J. (2010). Effects of Capecitabine and Celecoxib in Experimental Pancreatic Cancer. Pancreatol. Off. J. Int. Assoc. Pancreatol. IAP Al.

[8] Muthu R., Vaiyapuri M. (2013). Synergistic and Individual Effects of Umbelliferone with 5-Fluorouracil on Tumor Markers and Antioxidant Status of Rat Treated with 1,2-Dimethylhydrazine. Biomed. Aging Pathol..

[9] Patial V.S.M., Sharma S., Pratap K., Singh D., Padwad Y.S. (2015). Synergistic Effect of Curcumin and Piperine in Suppression of DENA-Induced Hepatocellular Carcinoma in Rats. Environ. Toxicol. Pharmacol..

[10] Habs M., Schmähl D. (1980). Synergistic Effects of N-Nitroso Compounds in Experimental Long-Term Carcinogenesis Studies. Oncology.

[11] Nishizumi M. (1976). Enhancement of Diethylnitrosamine Hepatocarcinogenesis in Rats by Exposure to Polychlorinated Biphenyls or Phenobarbital. Cancer Lett..

[12] Takayama S., Imaizumi T. (1969). Sequential Effects of Chemically Different Carcinogens, Dimethylnitrosamine and 4-Dimethylaminoazobenzene, on Hepatocarcinogenesis in Rats. Int. J. Cancer.

[13] Qian G., Tang L., Lin S., Xue K.S., Mitchell N.J., Su J., Gelderblom W.C., Riley R.T., Phillips T.D., Wang J.-S. (2016). Sequential Dietary Exposure to Aflatoxin B1 and Fumonisin B1 in F344 Rats Increases Liver Preneoplastic Changes Indicative of a Synergistic Interaction. Food Chem. Toxicol. Int. J. Publ. Br. Ind. Biol. Res. Assoc..

[14] Sekijima M., Tsutsumi T., Yoshida T., Harada T., Tashiro F., Chen G., Yu S.Z., Ueno Y. (1999). Enhancement of Glutathione S-Transferase Placental-Form Positive Liver Cell Foci Development by Microcystin-LR in Aflatoxin B1-Initiated Rats. Carcinogenesis.

[15] Song K.Y., Lim I.K., Park S.C., Lee S.O., Park H.S., Choi Y.K., Hyun B.H. (1999). Effect of Nodularin on the Expression of Glutathione S-Transferase Placental Form and Proliferating Cell Nuclear Antigen in N-Nitrosodiethylamine Initiated Hepatocarcinogenesis in the Male Fischer 344 Rat. Carcinogenesis.

[16] Shimo T., Mitsumori K., Onodera H., Yasuhara K., Kitaura K., Takahashi M., Kanno J., Hayashi Y. (1994). Synergistic Effects of Phenobarbital and Thiourea on Proliferative Lesions in the Rat Liver. Cancer Lett..

[17] Terao K., Aikawa T., Kera K. (1978). A Synergistic Effect of Nitrosodimethylamine on Sterigmatocystin Carcinogenesis in Rats. Food Cosmet. Toxicol..

[18] Gelderblom W.C., Snyman S.D., Lebepe-Mazur S., van der Westhuizen L., Kriek N.P., Marasas W.F. (1996). The Cancer-Promoting Potential of Fumonisin B1 in Rat Liver Using Diethylnitrosamine as a Cancer Initiator. Cancer Lett..

[19] Furuya K., Williams G.M. (1984). Neoplastic Conversion in Rat Liver by the Antihistamine Methapyrilene Demonstrated by a Sequential Syncarcinogenic Effect with N-2-Fluorenylacetamide. Toxicol. Appl. Pharmacol..

[20] Mochizuki Y., Furukawa K., Sawada N. (1983). Effect of Simultaneous Administration of Clofibrate with Diethylnitrosamine on Hepatic Tumorigenesis in the Rat. Cancer Lett..

[21] Schwarz M., Bannasch P., Kunz W. (1983). The Effect of Pre- and Post-Treatment with Phenobarbital on the Extent of Gamma-Glutamyl Transpeptidase Positive Foci Induced in Rat Liver by N-Nitrosomorpholine. Cancer Lett..

[22] Cho K.J., Jang J.J. (1993). Effects of Carbon Tetrachloride, Ethanol and Acetaldehyde on Diethylnitrosamine-Induced Hepatocarcinogenesis in Rats. Cancer Lett..

[23] Pound A.W., Lawson T.A., Horn L. (1973). Increased Carcinogenic Action of Dimethylnitrosamine after Prior Administration of Carbon Tetrachloride. Br. J. Cancer.

[24] Schmähl D., Krüger F.W., Habs M., Diehl B. (1976). Influence of Disulfiram on the Organotropy of the Carcinogenic Effect of Dimethylnitrosamine and Diethylnitrosamine in Rats. Z. Krebsforsch. Klin. Onkol. Cancer Res. Clin. Oncol..

[25] Hoch-Ligeti C., Argus M.F., Arcos J.C. (1968). Combined Carcinogenic Effects of Dimethylnitrosamine and 3-Methylcholanthrene in the Rat. J. Natl. Cancer Inst..

[26] Tsuda H., Miyata Y., Murasaki G., Kinoshita H., Fukushima S. (1977). Synergistic Effect of Urinary Bladder Carcinogenesis in Rats Treated with N-Butyl-n-(4-Hydroxybutyl)Nitrosamine, N-(4-(5-Nitro-2-Furyl)-2-Thiazolyl)Formamide,N-2-Fluorenylacetamide, and 3,3′-Dichlorobenzidine. Gan.

[27] Fujii T., Mikuriya H., Kamiya N., Hiraga K. (1986). Enhancing Effect of Thiabendazole on Urinary Bladder Carcinogenesis Induced by Sodium O-Phenylphenate in F344 Rats. Food Chem. Toxicol. Int. J. Publ. Br. Ind. Biol. Res. Assoc..

[28] Kurata Y., Asamoto M., Hagiwara A., Masui T., Fukushima S. (1986). Promoting Effects of Various Agents in Rat Urinary Bladder Carcinogenesis Initiated by N-Butyl-N-(4-Hydroxybutyl)Nitrosamine. Cancer Lett..

[29] Shirai K., Uemura Y., Fukumoto M., Tsukamoto T., Pascual R., Nandi S., Tsubura A. (1997). Synergistic Effect of MNU and DMBA in Mammary Carcinogenesis and H-Ras Activation in Female Sprague-Dawley Rats. Cancer Lett..

[30] Verdeal K., Ertürk E., Rose D.P. (1983). Effects of Reserpine Administration on Rat Mammary Tumors and Uterine Disease Induced by N-Nitrosomethylurea. Eur. J. Cancer Clin. Oncol..

[31] van Cauteren H., van den Berghe J., Hérin V., de Coster R., Marsboom R. (1984). Effect of a High Dosage of Ketoconazole All or Not Combined with Ovariectomy on N-Nitroso-N-Methylurea-Induced Mammary Cancer in the Rat. Cancer Lett..

[32] Appel M.J., van Garderen-Hoetmer A., Woutersen R.A. (1994). Effects of Dietary Linoleic Acid on Pancreatic Carcinogenesis in Rats and Hamsters. Cancer Res..

[33] Yaono M., Tamano S., Mori T., Kato K., Imaida K., Asamoto M., Shirai T. (2000). Lobe Specific Effects of Testosterone and Estrogen on 3,2′-Dimethyl-4-Aminobiphenyl-Induced Rat Prostate Carcinogenesis. Cancer Lett..

[34] Pollard M., Luckert P.H. (1987). Autochthonous Prostate Adenocarcinomas in Lobund-Wistar Rats: A Model System. Prostate.

[35] Chen H.-P., Pan M.-H., Chou Y.-Y., Sung C., Lee K.-H., Leung C.-M., Hsu P.-C. (2017). Effects of Di(2-Ethylhexyl)Phthalate Exposure on 1,2-Dimethyhydrazine-Induced Colon Tumor Promotion in Rats. Food Chem. Toxicol. Int. J. Publ. Br. Ind. Biol. Res. Assoc..

[36] Nakahara W., Fukuoka F. (1960). Summation of Carcinogenic Effects of Chemically Unrelated Carcinogens, 4-Nitroquinoline N-Oxide and 20-Methylcholanthrene. Gan.

[37] Wood A.W., Chang R.L., Huang M.T., Baggiolini E., Partridge J.J., Uskokovic M., Conney A.H. (1985). Stimulatory Effect of 1 Alpha, 25-Dihydroxyvitamin D3 on the Formation of Skin Tumors in Mice Treated Chronically with 7,12-Dimethylbenz[a]Anthracene. Biochem. Biophys. Res. Commun..

[38] Cardesa A., Pour P., Althoff J., Mohr U. (1974). Effects of Intraperitoneal Injections of Dimethyl- and Diethylnitrosamine, Alone or Simultaneously on Swiss Mice. Z. Krebsforsch. Klin. Onkol..

[39] Shirai T., Miyata Y., Nakanishi K., Murasaki G., Ito N. (1978). Hepatocarcinogenicity of Polychlorinated Terphenyl (PCT) in ICR Mice and Its Enhancement by Hexachlorobenzene (HCB). Cancer Lett..

[40] Montesano R., Saffiotti U., Ferrero A., Kaufman D.G. (1974). Synergistic Effects of Benzo(Alpha)Pyrene and Diethylnitrosamine on Respiratory Carcinogenesis in Hamsters. J. Natl. Cancer Inst..

[41] Kaufman D.G., Madison R.M., Karbe E., Park J.F. (1974). Synergistic Effects of Benzo(a)Pyrene and N-Methyl-N-Nitrosourea on Respiratory Carcinogenesis in Syrian Golden Hamsters. Experimental Lung Cancer: Carcinogenesis and Bioassays.

[42] Altuwairgi O.S., Papageorge M.B., Doku H.C. (1995). The Cancer-Promoting Effect of N-Nitrosonornicotine Used in Combination with a Subcarcinogenic Dose of 4-Nitroquinoline-N-Oxide and 7,12-Dimethylbenz (A) Anthracene. J. Oral Maxillofac. Surg. Off. J. Am. Assoc. Oral Maxillofac. Surg..

[43] Martins M., Santos J.M., Diniz M.S., Ferreira A.M., Costa M.H., Costa P.M. (2015). Effects of Carcinogenic versus Non-Carcinogenic AHR-Active PAHs and Their Mixtures: Lessons from Ecological Relevance. Environ. Res..

[44] Zhang L., Zhao R., Ye S.Q., Zhou L., Wu Y.N., Zeng Y. (2013). Synergistic Effects of 2,3,7,8-Tetrachlorodibenzo-p-Dioxin and N-Nitrosodiethylamine on Cell Malignant Transformation. Biomed. Environ. Sci. BES.

[45] Aboubakr E.M., Taye A., Aly O.M., Gamal-Eldeen A.M., El-Moselhy M.A. (2017). Enhanced Anticancer Effect of Combretastatin A-4 Phosphate When Combined with Vincristine in the Treatment of Hepatocellular Carcinoma. Biomed. Pharmacother. Biomed. Pharmacother..

[46] Ohishi T., Kishimoto Y., Miura N., Shiota G., Kohri T., Hara Y., Hasegawa J., Isemura M. (2002). Synergistic Effects of (-)-Epigallocatechin Gallate with Sulindac against Colon Carcinogenesis of Rats Treated with Azoxymethane. Cancer Lett..

[47] Sharma S.H., Thulasingam S., Chellappan D.R., Chinnaswamy P., Nagarajan S. (2017). Morin and Esculetin Supplementation Modulates C-Myc Induced Energy Metabolism and Attenuates Neoplastic Changes in Rats Challenged with the Procarcinogen 1,2-Dimethylhydrazine. Eur. J. Pharmacol..

[48] Shi N., Riedl K.M., Schwartz S.J., Zhang X., Clinton S.K., Chen T. (2016). Efficacy Comparison of Lyophilised Black Raspberries and Combination of Celecoxib and PBIT in Prevention of Carcinogen-Induced Oesophageal Cancer in Rats. J. Funct. Foods.

[49] Kothari A., Borges A., Ingle A., Kothari L. (1997). Combination of Melatonin and Tamoxifen as a Chemoprophylaxis against N-Nitroso-N-Methylurea-Induced Rat Mammary Tumors. Cancer Lett..

[50] Chatterjee M., Janarthan M., Manivannan R., Rana A., Chatterjee M. (2010). Combinatorial Effect of Fish Oil (Maxepa) and 1alpha,25-Dihydroxyvitamin D(3) in the Chemoprevention of DMBA-Induced Mammary Carcinogenesis in Rats. Chem. Biol. Interact..

[51] Alobaedi O.H., Talib W.H., Basheti I.A. (2017). Antitumor Effect of Thymoquinone Combined with Resveratrol on Mice Transplanted with Breast Cancer. Asian Pac. J. Trop. Med..

[52] Qureshi W.A., Zhao R., Wang H., Ji T., Ding Y., Ihsan A., Mujeeb A., Nie G., Zhao Y. (2016). Co-Delivery of Doxorubicin and Quercetin via MPEG–PLGA Copolymer Assembly for Synergistic Anti-Tumor Efficacy and Reducing Cardio-Toxicity. Sci. Bull..

[53] Wang Q., Wieder R. (2004). All-Trans Retinoic Acid Potentiates Taxotere-Induced Cell Death Mediated by Jun N-Terminal Kinase in Breast Cancer Cells. Oncogene.

[54] Wang Y., He Q.-Y., Chen H., Chiu J.-F. (2007). Synergistic Effects of Retinoic Acid and Tamoxifen on Human Breast Cancer Cells: Proteomic Characterization. Exp. Cell Res..

[55] Sun R., Liu Y., Li S.-Y., Shen S., Du X.-J., Xu C.-F., Cao Z.-T., Bao Y., Zhu Y.-H., Li Y.-P. (2015). Co-Delivery of All-Trans-Retinoic Acid and Doxorubicin for Cancer Therapy with Synergistic Inhibition of Cancer Stem Cells. Biomaterials.

[56] Grunt T., Dittrich E., Offterdinger M., Schneider S.M., Dittrich C., Huber H. (1998). Effects of Retinoic Acid and Fenretinide on the C-ErbB-2 Expression, Growth and Cisplatin Sensitivity of Breast Cancer Cells. Br. J. Cancer.

[57] Tian Y., Liu G., Wang H., Tian Z., Cai Z., Zhang F., Luo Y., Wang S., Guo G., Wang X. (2017). Valproic Acid Sensitizes Breast Cancer Cells to Hydroxyurea through Inhibiting RPA2 Hyperphosphorylation-Mediated DNA Repair Pathway. DNA Repair.

[58] Hu B., Sun D., Sun C., Sun Y.-F., Sun H.-X., Zhu Q.-F., Yang X.-R., Gao Y.-B., Tang W.-G., Fan J. (2015). A Polymeric Nanoparticle Formulation of Curcumin in Combination with Sorafenib Synergistically Inhibits Tumor Growth and Metastasis in an Orthotopic Model of Human Hepatocellular Carcinoma. Biochem. Biophys. Res. Commun..

[59] Lopez Lopez R., van Rijswijk R.E., Wagstaff J., Pinedo H.M., Peters G.J. (1994). The Synergistic and Antagonistic Effects of Cytotoxic and Biological Agents on the in Vitro Antitumour Effects of Suramin. Eur. J. Cancer.

[60] Wu X., Huang Z., Liu J., Chen Y., Huang H., He Y., Li D., Zhang L., Du Z., Zhang K. (2019). Effects and Mechanism of Inhibition of Naringin in Combination with Atorvastatin on Prostate Cancer Cells in Vitro and in Vivo. Phytochem. Lett..

[61] Spingarn A., Sacks P.G., Kelley D., Dannenberg A.J., Schantz S.P. (1998). Synergistic Effects of 13-Cis Retinoic Acid and Arachidonic Acid Cascade Inhibitors on Growth of Head and Neck Squamous Cell Carcinoma in Vitro. Otolaryngol.—Head Neck Surg..

[62] Scambia G., Ranelletti F.O., Benedetti Panici P., Piantelli M., Bonanno G., De Vincenzo R., Ferrandina G., Maggiano N., Capelli A., Mancuso S. (1992). Inhibitory Effect of Quercetin on Primary Ovarian and Endometrial Cancers and Synergistic Activity with Cis-Diamminedichloroplatinum (II). Gynecol. Oncol..

[63] Yabasin I.B., Ibrahim M.M., Adam A., Wilfred S.-A., Ziem J.B., Gao P., Kampo S., Qingping W. (2014). Anticancer Effects of Vecuronium Bromide and Cisatracurium Besylate on Lung Cancer Cells (A549), in Vitro. Biomed. Aging Pathol..

[64] Ding K., Lu L., Wang J., Wang J., Zhou M., Zheng C., Liu J., Zhang C., Zhuang S. (2017). In Vitro and in Silico Investigations of the Binary-Mixture Toxicity of Phthalate Esters and Cadmium (II) to Vibrio Qinghaiensis Sp.-Q67. Sci. Total Environ..

[65] Lin Z., Yu H., Wei D., Wang G., Feng J., Wang L. (2002). Prediction of Mixture Toxicity with Its Total Hydrophobicity. Chemosphere.

[66] Walker N.J., Crockett P.W., Nyska A., Brix A.E., Jokinen M.P., Sells D.M., Hailey J.R., Easterling M., Haseman J.K., Yin M. (2005). Dose-Additive Carcinogenicity of a Defined Mixture of “Dioxin-like Compounds”. Environ. Health Perspect..

[67] Hauschild V.D. (2000). Chemical Exposure Guidelines for Deployed Military Personnel. Drug Chem. Toxicol..

[68] National Toxicology Program: 14th Report on Carcinogens. https://ntp.niehs.nih.gov/go/roc14.

[69] List of Classifications—IARC Monographs Volume 1 to 125 on the Identification of Carcinogenic Hazards to Humans. https://monographs.iarc.who.int/list-of-classifications.

[70] The Japan Society for Occupational Health (2018). Recommendation of Occupational Exposure Limits (2018–2019). J. Occup. Health.

[71] Carcinogenic Potency Database. https://www.nlm.nih.gov/databases/download/cpdb.html.

[72] Godden J.W., Xue L., Bajorath J. (2000). Combinatorial preferences affect molecular similarity/diversity calculations using binary fingerprints and Tanimoto coefficients. J. Chem. Inf. Comput. Sci..

[73] Read-Across Assessment Framework. https://echa.europa.eu/documents/10162/13628/raaf_en.pdf/614e5d61-891d-4154-8a47-87efebd1851a.

[74] Moriwaki H., Tian Y.-S., Kawashita N., Takagi T. (2018). Mordred: A Molecular Descriptor Calculator. J. Cheminform..

[75] Gaudin T., Rotureau P., Fayet G. (2015). Mixture Descriptors toward the Development of Quantitative Structure–Property Relationship Models for the Flash Points of Organic Mixtures. Ind. Eng. Chem. Res..

[76] Limbu S., Zakka C., Dakshanamurthy S. (2022). Predicting Dose-Range Chemical Toxicity using Novel Hybrid Deep Machine-Learning Method. Toxics.

[77] Limbu S., Dakshanamurthy S. (2022). Predicting Chemical Carcinogens Using a Hybrid Neural Network Deep Learning Method. Sensors.

[78] Limbu S., Dakshanamurthy S. (2022). A New Hybrid Neural Network Deep Learning Method for Protein-Ligand Binding Affinity Prediction and De Novo Drug Design. Int. J. Mol. Sci..

[79] Goodson W.H., Lowe L., Carpenter D.O., Gilbertson M., Manaf Ali A., Lopez de Cerain Salsamendi A., Lasfar A., Carnero A., Azqueta A., Amedei A. (2015). Assessing the Carcinogenic Potential of Low-Dose Exposures to Chemical Mixtures in the Environment: The Challenge Ahead. Carcinogenesis.

[80] Miller M.F., Goodson W.H., Manjili M.H., Kleinstreuer N., Bisson W.H., Lowe L. (2017). Low-Dose Mixture Hypothesis of Carcinogenesis Workshop: Scientific Underpinnings and Research Recommendations. Environ. Health Perspect..

