# Supplementary material for: Predicting Dose-Dependent Carcinogenicity of Chemical Mixtures Using a Novel Hybrid Neural Network Framework and Mathematical Approach
Source: Toxics. 2023 Jul 12;11(7):605. doi: 10.3390/toxics11070605 (PMC10383376; doi:10.3390/toxics11070605)
Supplement: Supplementary file 1 [file toxics-11-00605-s001.zip › toxics-2452577-supplementary.pdf]

Supplementary Materials

Table S1. 54 Carcinogenic binary chemical combinations collected from the literature.

Species		Chemical 1	Chemical 2	Sources
Rat	liver	Diethylnitrosamine (DEN)	4-dimethylaminoazobenzene	Habs & Schmähl, 1980[1]
			4-dimethylaminostilbene	
	liver	Diethylnitrosamine (DEN)	phenobarbital	Nishizumi, 1976[2]
	liver	Dimethylnitrosamine (DMN)	4-dimethylaminoazobenzene (DAB)	Takayama & Imaizumi, 1969[3]
	liver	Aflatoxin B_1	Fumonisin B_1	Qian et al, 2016[4]
	liver	Diethylnitrosamine (DEN)	Aflatoxin B_1	Sekijima et al, 1999[5]
		Microcystin-LR		
	liver	Diethylnitrosamine (DEN)	Nodularin (NOD)	Song et al, 1999[6]
	Liver	n-bis(2-hyrdoxypropyl) nitrosamine (DHPN)	Phenobarbital (PB)	Shimo et al, 1994[7]
			Thiourea (TU)	
	Liver	Sterigmatocystin (STG)	Nitrosodimethylamine (NDMA)	Terao et al, 1978[8]
	Liver	Diethylnitrosamine (DEN)	Fumonisin B_1	Gelderblom et al, 1996[9]
	liver	N-2-fluorenylacetamide (2-FAA)	Methapyrilene (MP)	Furuya & Williams, 1984[10]
			Diethylnitrosamine (DEN)	
	liver	Diethylnitrosamine (DEN)	Clofibrate	Mochizuki et al, 1983[11]
	liver	N-nitrosomorphine (NNM)	Phenobarbital	Schwarz et al, 1983[12]
	liver	Diethylnitrosamine (DEN)	Carbon tetrachloride	Cho & Jang, 1993[13]
	liver and kidney	Dimethylnitrosamine (DMN)	carbon tetrachloride	Pound et al, 1973[14]
	Liver and squamous cell	Dimethylnitrosamine (DMNA)	disulfiram (DSF)	Schmähl et al, 1976[15]
		Diethylnitrosamine (DEN)	disulfiram (DSF)	
	lung	Dimethylnitrosamine (DMN)	3-methylcholanthrene (MCA)	Hoch-Ligeti et al, 1968[16]
	Lung and heart	Methyl-acetoxymethyl-nitrosamine	disulfiram (DSF)	Habs & Schmähl, 1980[1]

	urinary bladder	N-butyl-N-(4-hydroxybutyl) nitrosamine (BBN)	N-[4-(5-nitro-2-furyl)-2-thiazolyl] formamide (FANFT)	Tsuda et al, 1977[17]
		N-butyl-N-(4-hydroxybutyl) nitrosamine (BBN)	N-2-fluorenylacetamide (2-FAA)	
		N-butyl-N-(4-hydroxybutyl) nitrosamine (BBN)	3, 3'-dichlorobenzidine (3, 3'-DCB)	
		N-2-fluorenylacetamide (2-FAA)	N-[4-(5-nitro-2-furyl)-2-thiazolyl] formamide (FANFT)	
	Urinary bladder	Sodium o-phenylphenate (OPP-Na)	Thiabendazole (TBZ)	Fujii et al, 1986[18]
	Urinary bladder	n-butyl-n-(4-hydroxybutyl) nitrosamine(BBN)	diphenyl	Kurata et al, 1986[19]
	Mammary	Methylnitrosourea (MNU)	Estrone (E1)	Bigsby, 2002[20]
	mammary	N-methyl-N-nitrosourea (MNU)	7,12-dimethylbenz[α]anthracene (DMBA)	Shirai et al, 1997[21]
	mammary	N-nitrosomethylurea (NMU)	reserpine	Verdeal et al, 1983[22]
	mammary	N-nitrosomethylurea (NMU)	ketoconazole	Van Cauteren et al, 1984[23]
	Pancreatic	Azaserine	Linoleic acid (LA)	Appel et al, 1994[24]
	Prostate	3,2'-dimethyl-4-aminobiphenyl (DMAB)	Testosterone propionate (TP)	Yaono et al, 2000[25]
	prostate	N-mthyl-N-nitrosourea (MNU)	Testosterone propionate (TP)	Pollard & Luckert, 1987[26]
	Colon	di(2-ethylhexyl)phthalate (DEHP)	1,2-dimethylhydrazine (DMH)	Chen et al, 2017[27]
Mouse	skin	4-nitroquinolone n-oxide	20-methylcholanthrene	Nakahara & Fukuoka, 1960[28]
	skin	7,12-dimethylbenz[α]anthracene (DMBA)	1α,25-dihydroxyvitamin D3	Wood et al, 1985[29]
	lung	Dimethylnitrosamine (DMN)	Diethylnitrosamine (DEN)	Cardesa et al, 1974[30]
	liver	Polychlorinated terphenyl (PCT)	Hexachlorobenzene (HCB)	Shirai et al, 1978[31]
Hamster	Squamous cell	Diethylnitrosamine (DEN)	Benzo[a]pyrene (BaP)	Montesano et al, 1974[32]
	Squamous cell	N-methyl-N-nitrosourea (MNU)	Benzo[a]pyrene (BaP)	Kaufman & Madison, 1974[33]

	Squamous cell	N-nitrosonornicotine (NNN)	7,12 dimethylbenz[α]anthracene (DMBA)	Altuwairgi et al, 1995[34]
			4-nitroquinolone n-oxide	
Fish	liver	N-methyl-N'-nitro-nitrosoguanidine (MNNG)	Fumonisin B1	Carlson et al, 2001[35]
	liver	Aflatoxin B_1	Tocopheryl acetate	Adams & Whitten, 2018[36]
	Histopathological Analysis	Benzo[b]fluoranthene (BBF)	Phenanthrene	Martins et al, 2015[37]
HepG2 Cells	DNA adduct formation	Benzo[a]pyrene (BaP)	Dibenzo[a,h]anthracene (DB[a,h]A)	Staal et al, 2007[38]
			Benzo[b]fluoranthene (B[b]F)	
			Fluoranthene (FA)	
			1-methylphenanthrene (1-MPA)	
			Dibenzo[a,l]pyrene (DB[a,l]P)	
HepG2/WB-F344 liver cell lines	DNA adduct formation	Benzo[a]pyrene (BaP)	7H-dibenzo[c,g]carbazole (DBC)	Gábelova et al, 2013[39]
Balb/c 3T3 cells	Cell transformation frequency	Diethylnitrosamine (DEN)	2,3,7,8-Tetracholorodibenzo-p-dioxin (TCDD)	Zhang et al, 2013[40]

Table S2. 25 non-carcinogenic binary drug and chemical combinations collected from the literature.

**Species**		**Chemical 1**	**Chemical 2**	**Sources**
Rat	liver	Curcumin	Piperine	Patial et al, 2015[41]
	liver	Combretastatin A-4 phosphate (CA4-P)	Vincristine	Aboubakr et al, 2017[42]
	colon	Umbelliferone (UMB)	5-fluorouracil (5-FU)	Muthu & Vaiyapuri, 2013[43]
	colon	(-)-Epigallocatechin gallate (EGCG)	Sulindac	Ohishi et al, 2002[44]
	colon	morin	esculetin	Sharma et al, 2017[45]
	oesophageal	Celecoxib	S,S'-1,4-phenylene-bis(1,2-ethanediyl)bis-isothiourea (PBIT)	Shi et al, 2016[46]
	mammary	Melatonin	Tamoxifen	Kothari et al, 1997[47]
	Mammary	Fish oil (Maxepa)	1α,25-dihydroxyvitamin D_3	Chatterjee et al, 2010[48]
mouse	Breast cancer	Thymoquinone (TQ)	Resveratrol (RES)	Alobaedi et al, 2017[49]

hamster	Pancreatic cancer	Celecoxib	capecitabine	Arjona-Sánchez et al, 2010[50]
Cell line	Breast cancer	Doxorubicin	Quercetin	Qureshi et al, 2016[51]
	Breast cancer	All-trans retinoic acid (ATRA)	Docetaxel (Taxotere)	Wang & Wieder, 2004[52]
	Breast cancer	All-trans retinoic acid (ATRA)	Tamoxifen	Wang et al, 2007[53]
	Breast cancer	All-trans retinoic acid (ATRA)	Doxorubicin	Sun et al, 2015[54]
	Breast cancer	All-trans retinoic acid (ATRA)	Cisplatin (CDDP)	Grunt et al, 1998[55]
	Breast cancer	Valproic acid	Hydroxyurea (HU)	Tian et al, 2017[56]
	Hepatocellular carcinoma	Curcumin	Sorafenib	Hu et al, 2015[57]
	Prostate cancer	Suramin	Cisplatin (CDDP)	Lopez Lopez et al, 1994[58]
	Prostate cancer	Naringin	Atorvastatin	Wu et al, 2019[59]
	Head and neck squamous cell	13-cis retinoic acid	Curcumin	Spingarn et al, 1998[60]
			phenidone	
			Nordihydroguaiaretic acid (NDGA)	
			5,8,11,14-eicosatetraynoic acid (ETYA)	
	Ovary and endometrial cancer	Cisplatin (CDDP)	Quercetin	Scambia et al, 1992[61]
	Lung cancer	Cisplatin (CDDP)	Cisatracurium besylate	Yabasin et al, 2014[62]

Table S3. Change in % accuracy for distinct training and test sets of virtual mixtures for the binary classification to predict carcinogenicity of chemical mixtures.

Classification		Accuracy %		% change
		Train/Test Not Separated	Train/Test Separated	
Binary	HNN	98.86	72.91	26.25
	RF	98.71	88	10.85
	Bag	98.3	87.39	11.10
	Ada	99.78	98.16	1.62

Table S4. Change in % accuracy for distinct training and test sets of virtual mixtures for the multiclass classification to predict carcinogenicity of chemical mixtures.

Classification		Accuracy %		% change
		Train/Test Not Separated	Train/Test Separated	
Multiclass	HNN	96.03	54.65	43.09
	RF	91.39	62.22	31.92
	Bag	91.74	58.17	36.59
	Ada	80.21	51.25	36.11

Table S5. Change in coefficient of determination (R^2) for distinct training and test sets of virtual mixtures for the regression classification to predict carcinogenic potency of chemical mixtures.

Classification		Coefficient of determination (R^2)		% change
		Train/Test Not Separated	Train/Test Separated	
Regression	HNN	0.962	0.383	60.18
	RF	0.903	0.285	68.44
	SVM	0.777	-0.147	118.92
	GB	0.942	0.251	73.35
	KR	0.961	0.036	96.25
	DTBoost	0.961	0.202	78.98
	NN	0.97	-0.448	146.19
	Consensus	0.959	0.229	76.12

Equation S1. Equations to calculate the evaluation metrics for binary & multiclass classification.

$$Accuracy = \frac{TP + TN}{TP + TN + FN + FP} \times 100$$

$$Sensitivity(truepositiverate) = \frac{TP}{TP + FN} \times 100$$

$$Specificity(truenegativerate) = \frac{TN}{TN + FP} \times 100$$

Where, TP = True Positive, TN = True Negative, FP = False Positive, and FN = False Negative.

Equation S2. Equations to calculate the evaluation metrics for regression models.

$$R^2 = \frac{ESS}{TSS} = \frac{\sum_{i=1}^{n}(\hat{y}_i - \bar{y})^2}{\sum_{i=1}^{n}(y_i - \bar{y})^2}$$

$$MSE = \frac{\sum_{i=1}^{n} |\hat{y}_i - y_i|^2}{n}$$

$$MAE = \frac{\sum_{i=1}^{n} |\hat{y}_i - y_i|}{n}$$

Where, ESS is explained sum of squares and TSS is the total sum of squares, $\hat{y}_i$ is the predicted value of the ith dependent variable, y_i is the ith observed dependent variable, and $\bar{y}$ is the mean of the observed data.

The number of unique simulations were run is 30 and average is taken for each statistical metrics such as AUC, accuracy, selectivity, sensitivity and precision.

In the context of the case 3 assumption scenario for creating a virtual binary mixture, the description of how two chemicals that are non-carcinogenic can result in a carcinogenic mixture, are provided below.

It is possible that two non-carcinogenic chemicals combine to form a carcinogenic mixture under certain specific conditions or mechanisms of exposure. There are several mechanisms by which this can occur, such as the following:

1. When two non-carcinogenic chemicals are combined, their combined effect is greater than the sum of their individual effects. This enhanced effect could lead to cellular damage or mutations that may promote carcinogenesis. For example, individually, the two non-carcinogenic chemicals may not cause significant DNA damage. However, when combined, they might interact in a way that increases the production of reactive oxygen species or other harmful reactive substances, leading to higher levels of DNA damage and an increased risk of cancer.
2. Noncarcinogenic chemicals may have differential modes or mechanisms of action, however, when they are combined, their toxic effects may be amplified. This can lead to damage to cells and tissues, potentially promoting the development of cancer.
3. In another scenario, in non-carcinogenic chemical combinations, one chemical may interfere with the cell's ability to repair DNA damage caused by the other chemical, resulting in the accumulation of mutations and become carcinogenic.
4. A non-carcinogenic chemical may augment the absorption or accumulation of another noncarcinogenic chemical in a co-exposure scenario, leading to higher exposure levels than would occur with either chemical alone. Such increased exposure can increase the possibility of DNA damage or mutation, and potentially carcinogenic.
5. Chemicals can act as procarcinogens, meaning they are not directly carcinogenic themselves but can be converted into carcinogens through metabolic activation to become carcinogenic. Individually, these chemicals are not toxic or have low carcinogenic potential, however, when these two non-carcinogenic chemicals are combined, these procarcinogens may undergo metabolic reactions and activation that result in reactive intermediates or DNA-damaging compounds, leading to carcinogenesis.
6. Combining two non-carcinogenic chemicals may disrupt normal biological signaling pathways or cellular genetic and epigenetic processes, leading to the activation of oncogenes or the inactivation of tumor suppressor genes. These alterations can promote cell proliferation or cell death leading to an increased risk of cancer.

The difference between case 4 and case 5 assumption scenario for creating a virtual binary mixture.

Case 4: The first chemical is carcinogenic, and the second chemical is non-carcinogenic.

Case 5: The first chemical is noncarcinogenic, and the second chemical is carcinogenic.

In cases 4 and 5, the following two scenarios may happen.

In the first scenario, if the first chemical in the mixture is carcinogenic, it can initiate or promote carcinogenesis. The noncarcinogenic second chemical, although not directly contributing to carcinogenesis, may still have other adverse effects or interactions. These effects could include enhancing the absorption, metabolism, or distribution of the carcinogenic chemical, leading to increased exposure and potentially aggravating its carcinogenic properties. Additionally, the second noncarcinogenic chemical may influence the progression or severity of cancer initiated by the first carcinogenic chemical by affecting the cellular signaling or modulating the cellular responses or DNA repair mechanisms.

In the second scenario, if the first chemical in the mixture is noncarcinogenic, it will not initiate or promote carcinogenesis on its own. However, the second carcinogenic chemical may interact with the noncarcinogenic chemical, potentially enhancing its carcinogenic properties. This interaction could involve increased absorption, distribution, or metabolism of the carcinogen due to the presence of the noncarcinogenic chemical. The first noncarcinogenic chemical might also affect cellular signaling or modulate the cellular responses or DNA repair mechanisms, which could influence the carcinogenic potential of the second chemical.

Both the first and second scenarios may happen for both cases 4 and 5.

These detailed descriptions are provided in the supplementary materials.

Multiple Mixtures

Multiple mixtures refer to the combination of two or more different chemical substances that are combined to form a mixture. The multiple mixtures can contain chemicals, metals, metalloids, and other air, water, and soil substances, and they are mixed together to form a homogeneous or heterogeneous mixture. The multiple mixtures are frequently present in industrial chemicals, environmental samples, household cleaners, personal care products, food, and beverages, etc.

Description of the classifications of Class 0, Class 1 and Class 2.

The chemicals are classified into different classes based on the different carcinogenic group classifications: Group A (Human Carcinogen), Group B (Probable human carcinogen), Group C (Possible human carcinogen), Group D (Not classifiable), and Group E (No evidence of carcinogenicity). These different groups are defined by the *Chemical Exposure Guidelines for Deployed Military Personnel Version 1.3 (MEG)*[13], *National Toxicology Program (NTP)*[14], *International Agency for Research on Cancer (IARC)*[15], and *The Japan Society for Occupational Health (JSOH)*[16]. As explained in Section II of the Methods Section, carcinogenic chemicals are classified into five distinct groups: Group A (Human Carcinogen), Group B (Probable human carcinogen), Group C (Possible human carcinogen), Group D (Not classifiable), and Group E (No evidence of carcinogenicity).

To reduce the complexity in our multiclassification, class 2 is formed by combining two group classes into one class that is Group A (Human Carcinogen) and Group B (Probable human carcinogen) are combined to form class 2 chemicals. Whereas Class 1 is formed, by combining two group classes into one class that is Group C

(Possible human carcinogen) and Group D (Not classifiable) are combined to form class 1 chemicals. Class 0 chemicals are classified as noncarcinogenic, which signifies Group E (No evidence of carcinogenicity). Though both classifications i.e. possibly carcinogenic (Class 1) and probable carcinogens (Class 2), imply a potential risk of cancer, with the "probable human carcinogen" category indicating a higher level of certainty based on available evidence, there are differences between these classes as below.

Possible human carcinogen group of chemicals indicates that there is limited evidence indicating a substance that can cause cancer in humans, but the available data is not sufficient to establish a definitive link. It means there are some studies or evidence suggesting a potential carcinogenic effect, however, additional research is needed to confirm the cancer association. Further investigation might be required to determine the strength and consistency of the evidence to cancer link.

Probable human carcinogen group of chemicals indicates that there is strong evidence indicating a substance that can cause cancer in humans, but there may still be some uncertainties. It means there is sufficient evidence from animal studies, demonstrating a carcinogenic effect, however, there might be limitations in the data, such as the strength of the association or the possibility of alternative explanations. Despite these uncertainties, the overall evidence is substantial enough to establish a probable link between the substance and cancer.

REFERENCES:

(1) Habs, M.; Schmähl, D. Synergistic Effects of N-Nitroso Compounds in Experimental Long-Term Carcinogenesis Studies. *Oncology* **1980**, *37* (4), 259–265. https://doi.org/10.1159/000225448.

(2) Nishizumi, M. Enhancement of Diethylnitrosamine Hepatocarcinogenesis in Rats by Exposure to Polychlorinated Biphenyls or Phenobarbital. *Cancer Lett.* **1976**, *2* (1), 11–15. https://doi.org/10.1016/s0304-3835(76)80004-3.

(3) Takayama, S.; Imaizumi, T. Sequential Effects of Chemically Different Carcinogens, Dimethylnitrosamine and 4-Dimethylaminoazobenzene, on Hepatocarcinogenesis in Rats. *Int. J. Cancer* **1969**, *4* (4), 373–383. https://doi.org/10.1002/ijc.2910040402.

(4) Qian, G.; Tang, L.; Lin, S.; Xue, K. S.; Mitchell, N. J.; Su, J.; Gelderblom, W. C.; Riley, R. T.; Phillips, T. D.; Wang, J.-S. Sequential Dietary Exposure to Aflatoxin B1 and Fumonisin B1 in F344 Rats Increases Liver Preneoplastic Changes Indicative of a Synergistic Interaction. *Food Chem. Toxicol. Int. J. Publ. Br. Ind. Biol. Res. Assoc.* **2016**, *95*, 188–195. https://doi.org/10.1016/j.fct.2016.07.017.

(5) Sekijima, M.; Tsutsumi, T.; Yoshida, T.; Harada, T.; Tashiro, F.; Chen, G.; Yu, S. Z.; Ueno, Y. Enhancement of Glutathione S-Transferase Placental-Form Positive Liver Cell Foci Development by Microcystin-LR in Aflatoxin B1-Initiated Rats. *Carcinogenesis* **1999**, *20* (1), 161–165. https://doi.org/10.1093/carcin/20.1.161.

(6) Song, K. Y.; Lim, I. K.; Park, S. C.; Lee, S. O.; Park, H. S.; Choi, Y. K.; Hyun, B. H. Effect of Nodularin on the Expression of Glutathione S-Transferase Placental Form and Proliferating Cell Nuclear Antigen in N-Nitrosodiethylamine Initiated Hepatocarcinogenesis in the Male Fischer 344 Rat. *Carcinogenesis* **1999**, *20* (8), 1541–1548. https://doi.org/10.1093/carcin/20.8.1541.

(7) Shimo, T.; Mitsumori, K.; Onodera, H.; Yasuhara, K.; Kitaura, K.; Takahashi, M.; Kanno, J.; Hayashi, Y. Synergistic Effects of Phenobarbital and Thiourea on Proliferative Lesions in the Rat Liver. *Cancer Lett.* **1994**, *81* (1), 45–52. https://doi.org/10.1016/0304-3835(94)90163-5.

(8) Terao, K.; Aikawa, T.; Kera, K. A Synergistic Effect of Nitrosodimethylamine on Sterigmatocystin Carcinogenesis in Rats. *Food Cosmet. Toxicol.* **1978**, *16* (6), 591–596. https://doi.org/10.1016/S0015-6264(78)80228-4.

(9) Gelderblom, W. C.; Snyman, S. D.; Lebepe-Mazur, S.; van der Westhuizen, L.; Kriek, N. P.; Marasas, W. F. The Cancer-Promoting Potential of Fumonisin B1 in Rat Liver Using Diethylnitrosamine as a Cancer Initiator. *Cancer Lett.* **1996**, *109* (1–2), 101–108. https://doi.org/10.1016/s0304-3835(96)04431-x.

(10) Furuya, K.; Williams, G. M. Neoplastic Conversion in Rat Liver by the Antihistamine Methapyrilene Demonstrated by a Sequential Syncarcinogenic Effect with N-2-Fluorenylacetamide. *Toxicol. Appl. Pharmacol.* **1984**, *74* (1), 63–69. https://doi.org/10.1016/0041-008x(84)90270-9.

(11) Mochizuki, Y.; Furukawa, K.; Sawada, N. Effect of Simultaneous Administration of Clofibrate with Diethylnitrosamine on Hepatic Tumorigenesis in the Rat. *Cancer Lett.* **1983**, *19* (1), 99–105. https://doi.org/10.1016/0304-3835(83)90142-8.

(12) Schwarz, M.; Bannasch, P.; Kunz, W. The Effect of Pre- and Post-Treatment with Phenobarbital on the Extent of Gamma-Glutamyl Transpeptidase Positive Foci Induced in Rat Liver by N-Nitrosomorpholine. *Cancer Lett.* **1983**, *21* (1), 17–21. https://doi.org/10.1016/0304-3835(83)90077-0.

(13) Cho, K. J.; Jang, J. J. Effects of Carbon Tetrachloride, Ethanol and Acetaldehyde on Diethylnitrosamine-Induced Hepatocarcinogenesis in Rats. *Cancer Lett.* **1993**, *70* (1–2), 33–39. https://doi.org/10.1016/0304-3835(93)90071-g.

(14) Pound, A. W.; Lawson, T. A.; Horn, L. Increased Carcinogenic Action of Dimethylnitrosamine after Prior Administration of Carbon Tetrachloride. *Br. J. Cancer* **1973**, *27* (6), 451–459. https://doi.org/10.1038/bjc.1973.57.

(15) Schmähl, D.; Krüger, F. W.; Habs, M.; Diehl, B. Influence of Disulfiram on the Organotropy of the Carcinogenic Effect of Dimethylnitrosamine and Diethylnitrosamine in Rats. *Z. Krebsforsch. Klin. Onkol. Cancer Res. Clin. Oncol.* **1976**, *85* (3), 271–276. https://doi.org/10.1007/BF00284085.

(16) Hoch-Ligeti, C.; Argus, M. F.; Arcos, J. C. Combined Carcinogenic Effects of Dimethylnitrosamine and 3-Methylcholanthrene in the Rat. *J. Natl. Cancer Inst.* **1968**, *40* (3), 535–549.

(17) Tsuda, H.; Miyata, Y.; Murasaki, G.; Kinoshita, H.; Fukushima, S. Synergistic Effect of Urinary Bladder Carcinogenesis in Rats Treated with N-Butyl-n-(4-Hydroxybutyl)Nitrosamine, N-(4-(5-Nitro-2-Furyl)-2-Thiazolyl)Formamide,N-2-Fluorenylacetamide, and 3,3'-Dichlorobenzidine. *Gan* **1977**, *68* (2), 183–192.
(18) Fujii, T.; Mikuriya, H.; Kamiya, N.; Hiraga, K. Enhancing Effect of Thiabendazole on Urinary Bladder Carcinogenesis Induced by Sodium O-Phenylphenate in F344 Rats. *Food Chem. Toxicol. Int. J. Publ. Br. Ind. Biol. Res. Assoc.* **1986**, *24* (3), 207–211. https://doi.org/10.1016/0278-6915(86)90230-9.
(19) Kurata, Y.; Asamoto, M.; Hagiwara, A.; Masui, T.; Fukushima, S. Promoting Effects of Various Agents in Rat Urinary Bladder Carcinogenesis Initiated by N-Butyl-N-(4-Hydroxybutyl)Nitrosamine. *Cancer Lett.* **1986**, *32* (2), 125–135. https://doi.org/10.1016/0304-3835(86)90110-2.
(20) Bigsby, R. M. Synergistic Tumor Promoter Effects of Estrone and Progesterone in Methylnitrosourea-Induced Rat Mammary Cancer. *Cancer Lett.* **2002**, *179* (2), 113–119. https://doi.org/10.1016/s0304-3835(02)00032-0.
(21) Shirai, K.; Uemura, Y.; Fukumoto, M.; Tsukamoto, T.; Pascual, R.; Nandi, S.; Tsubura, A. Synergistic Effect of MNU and DMBA in Mammary Carcinogenesis and H-Ras Activation in Female Sprague-Dawley Rats. *Cancer Lett.* **1997**, *120* (1), 87–93. https://doi.org/10.1016/s0304-3835(97)00293-0.
(22) Verdeal, K.; Ertürk, E.; Rose, D. P. Effects of Reserpine Administration on Rat Mammary Tumors and Uterine Disease Induced by N-Nitrosomethylurea. *Eur. J. Cancer Clin. Oncol.* **1983**, *19* (6), 825–834. https://doi.org/10.1016/0277-5379(83)90015-9.
(23) van Cauteren, H.; van den Berghe, J.; Hérin, V.; de Coster, R.; Marsboom, R. Effect of a High Dosage of Ketoconazole All or Not Combined with Ovariectomy on N-Nitroso-N-Methylurea-Induced Mammary Cancer in the Rat. *Cancer Lett.* **1984**, *24* (2), 193–200. https://doi.org/10.1016/0304-3835(84)90136-8.
(24) Appel, M. J.; van Garderen-Hoetmer, A.; Woutersen, R. A. Effects of Dietary Linoleic Acid on Pancreatic Carcinogenesis in Rats and Hamsters. *Cancer Res.* **1994**, *54* (8), 2113–2120.
(25) Yaono, M.; Tamano, S.; Mori, T.; Kato, K.; Imaida, K.; Asamoto, M.; Shirai, T. Lobe Specific Effects of Testosterone and Estrogen on 3,2'-Dimethyl-4-Aminobiphenyl-Induced Rat Prostate Carcinogenesis. *Cancer Lett.* **2000**, *150* (1), 33–40. https://doi.org/10.1016/s0304-3835(99)00370-5.
(26) Pollard, M.; Luckert, P. H. Autochthonous Prostate Adenocarcinomas in Lobund-Wistar Rats: A Model System. *The Prostate* **1987**, *11* (3), 219–227. https://doi.org/10.1002/pros.2990110303.
(27) Chen, H.-P.; Pan, M.-H.; Chou, Y.-Y.; Sung, C.; Lee, K.-H.; Leung, C.-M.; Hsu, P.-C. Effects of Di(2-Ethylhexyl)Phthalate Exposure on 1,2-Dimethyhydrazine-Induced Colon Tumor Promotion in Rats. *Food Chem. Toxicol. Int. J. Publ. Br. Ind. Biol. Res. Assoc.* **2017**, *103*, 157–167. https://doi.org/10.1016/j.fct.2017.03.014.
(28) Nakahara, W.; Fukuoka, F. Summation of Carcinogenic Effects of Chemically Unrelated Carcinogens, 4-Nitroquinoline N-Oxide and 20-Methylcholanthrene. *Gan* **1960**, *51*, 125–137.
(29) Wood, A. W.; Chang, R. L.; Huang, M. T.; Baggiolini, E.; Partridge, J. J.; Uskokovic, M.; Conney, A. H. Stimulatory Effect of 1 Alpha, 25-Dihydroxyvitamin D3 on the Formation of Skin Tumors in Mice Treated Chronically with 7,12-Dimethylbenz[a]Anthracene. *Biochem. Biophys. Res. Commun.* **1985**, *130* (2), 924–931. https://doi.org/10.1016/0006-291x(85)90505-4.
(30) Cardesa, A.; Pour, P.; Althoff, J.; Mohr, U. Effects of Intraperitoneal Injections of Dimethyl- and Diethylnitrosamine, Alone or Simultaneously on Swiss Mice. *Z. Für Krebsforsch. Klin. Onkol.* **1974**, *82* (3), 233–238. https://doi.org/10.1007/BF00304061.
(31) Shirai, T.; Miyata, Y.; Nakanishi, K.; Murasaki, G.; Ito, N. Hepatocarcinogenicity of Polychlorinated Terphenyl (PCT) in ICR Mice and Its Enhancement by Hexachlorobenzene (HCB). *Cancer Lett.* **1978**, *4* (5), 271–275. https://doi.org/10.1016/s0304-3835(78)95117-0.
(32) Montesano, R.; Saffiotti, U.; Ferrero, A.; Kaufman, D. G. Synergistic Effects of Benzo(Alpha)Pyrene and Diethylnitrosamine on Respiratory Carcinogenesis in Hamsters. *J. Natl. Cancer Inst.* **1974**, *53* (5), 1395–1397. https://doi.org/10.1093/jnci/53.5.1395.
(33) Kaufman, D. G.; Madison, R. M. Synergistic Effects of Benzo(a)Pyrene and N-Methyl-N-Nitrosourea on Respiratory Carcinogenesis in Syrian Golden Hamsters. In *Experimental Lung Cancer: Carcinogenesis and Bioassays*; Karbe, E., Park, J. F., Eds.; Springer: Berlin, Heidelberg, 1974; pp 207–218. https://doi.org/10.1007/978-3-642-61939-7_20.

(34) Altuwairgi, O. S.; Papageorge, M. B.; Doku, H. C. The Cancer-Promoting Effect of N-Nitrosonornicotine Used in Combination with a Subcarcinogenic Dose of 4-Nitroquinoline-N-Oxide and 7,12-Dimethylbenz (A) Anthracene. *J. Oral Maxillofac. Surg. Off. J. Am. Assoc. Oral Maxillofac. Surg.* **1995**, *53* (8), 910–913; discussion 914. https://doi.org/10.1016/0278-2391(95)90279-1.
(35) Carlson, D. B.; Williams, D. E.; Spitsbergen, J. M.; Ross, P. F.; Bacon, C. W.; Meredith, F. I.; Riley, R. T. Fumonisin B1 Promotes Aflatoxin B1 and N-Methyl-N'-Nitro-Nitrosoguanidine-Initiated Liver Tumors in Rainbow Trout. *Toxicol. Appl. Pharmacol.* **2001**, *172* (1), 29–36. https://doi.org/10.1006/taap.2001.9129.
(36) Adams, B. L.; Whitten, R. O. Hepatocellular Carcinomas Are Promoted by Tocopheryl Acetate but Eliminated by Tocopheryl Succinate. *J. Nutr. Intermed. Metab.* **2018**, *13*, 33–47. https://doi.org/10.1016/j.jnim.2018.07.006.
(37) Martins, M.; Santos, J. M.; Diniz, M. S.; Ferreira, A. M.; Costa, M. H.; Costa, P. M. Effects of Carcinogenic versus Non-Carcinogenic AHR-Active PAHs and Their Mixtures: Lessons from Ecological Relevance. *Environ. Res.* **2015**, *138*, 101–111. https://doi.org/10.1016/j.envres.2015.02.010.
(38) Staal, Y. C. M.; Hebels, D. G. A. J.; van Herwijnen, M. H. M.; Gottschalk, R. W. H.; van Schooten, F. J.; van Delft, J. H. M. Binary PAH Mixtures Cause Additive or Antagonistic Effects on Gene Expression but Synergistic Effects on DNA Adduct Formation. *Carcinogenesis* **2007**, *28* (12), 2632–2640. https://doi.org/10.1093/carcin/bgm182.
(39) Gábelová, A.; Poláková, V.; Prochazka, G.; Kretová, M.; Poloncová, K.; Regendová, E.; Luciaková, K.; Segerbäck, D. Sustained Induction of Cytochrome P4501A1 in Human Hepatoma Cells by Co-Exposure to Benzo[a]Pyrene and 7H-Dibenzo[c,g]Carbazole Underlies the Synergistic Effects on DNA Adduct Formation. *Toxicol. Appl. Pharmacol.* **2013**, *271* (1), 1–12. https://doi.org/10.1016/j.taap.2013.04.016.
(40) Zhang, L.; Zhao, R.; Ye, S. Q.; Zhou, L.; Wu, Y. N.; Zeng, Y. Synergistic Effects of 2,3,7,8-Tetrachlorodibenzo-p-Dioxin and N-Nitrosodiethylamine on Cell Malignant Transformation. *Biomed. Environ. Sci. BES* **2013**, *26* (5), 323–330. https://doi.org/10.3967/0895-3988.2013.05.001.
(41) Patial, V.; S, M.; Sharma, S.; Pratap, K.; Singh, D.; Padwad, Y. S. Synergistic Effect of Curcumin and Piperine in Suppression of DENA-Induced Hepatocellular Carcinoma in Rats. *Environ. Toxicol. Pharmacol.* **2015**, *40* (2), 445–452. https://doi.org/10.1016/j.etap.2015.07.012.
(42) Aboubakr, E. M.; Taye, A.; Aly, O. M.; Gamal-Eldeen, A. M.; El-Moselhy, M. A. Enhanced Anticancer Effect of Combretastatin A-4 Phosphate When Combined with Vincristine in the Treatment of Hepatocellular Carcinoma. *Biomed. Pharmacother. Biomedecine Pharmacother.* **2017**, *89*, 36–46. https://doi.org/10.1016/j.biopha.2017.02.019.
(43) Muthu, R.; Vaiyapuri, M. Synergistic and Individual Effects of Umbelliferone with 5-Fluorouracil on Tumor Markers and Antioxidant Status of Rat Treated with 1,2-Dimethylhydrazine. *Biomed. Aging Pathol.* **2013**, *3* (4), 219–227. https://doi.org/10.1016/j.biomag.2013.08.007.
(44) Ohishi, T.; Kishimoto, Y.; Miura, N.; Shiota, G.; Kohri, T.; Hara, Y.; Hasegawa, J.; Isemura, M. Synergistic Effects of (-)-Epigallocatechin Gallate with Sulindac against Colon Carcinogenesis of Rats Treated with Azoxymethane. *Cancer Lett.* **2002**, *177* (1), 49–56. https://doi.org/10.1016/s0304-3835(01)00767-4.
(45) Sharma, S. H.; Thulasingam, S.; Chellappan, D. R.; Chinnaswamy, P.; Nagarajan, S. Morin and Esculetin Supplementation Modulates C-Myc Induced Energy Metabolism and Attenuates Neoplastic Changes in Rats Challenged with the Procarcinogen 1,2 - Dimethylhydrazine. *Eur. J. Pharmacol.* **2017**, *796*, 20–31. https://doi.org/10.1016/j.ejphar.2016.12.019.
(46) Shi, N.; Riedl, K. M.; Schwartz, S. J.; Zhang, X.; Clinton, S. K.; Chen, T. Efficacy Comparison of Lyophilised Black Raspberries and Combination of Celecoxib and PBIT in Prevention of Carcinogen-Induced Oesophageal Cancer in Rats. *J. Funct. Foods* **2016**, *27*, 84–94. https://doi.org/10.1016/j.jff.2016.08.044.
(47) Kothari, A.; Borges, A.; Ingle, A.; Kothari, L. Combination of Melatonin and Tamoxifen as a Chemoprophylaxis against N-Nitroso-N-Methylurea-Induced Rat Mammary Tumors. *Cancer Lett.* **1997**, *111* (1–2), 59–66. https://doi.org/10.1016/s0304-3835(96)04493-x.
(48) Chatterjee, M.; Janarthan, M.; Manivannan, R.; Rana, A.; Chatterjee, M. Combinatorial Effect of Fish Oil (Maxepa) and 1alpha,25-Dihydroxyvitamin D(3) in the Chemoprevention of DMBA-Induced Mammary

Carcinogenesis in Rats. *Chem. Biol. Interact.* **2010**, *188* (1), 102–110. https://doi.org/10.1016/j.cbi.2010.06.007.
(49) Alobaedi, O. H.; Talib, W. H.; Basheti, I. A. Antitumor Effect of Thymoquinone Combined with Resveratrol on Mice Transplanted with Breast Cancer. *Asian Pac. J. Trop. Med.* **2017**, *10* (4), 400–408. https://doi.org/10.1016/j.apjtm.2017.03.026.
(50) Arjona-Sánchez, A.; Ruiz-Rabelo, J.; Perea, M. D.; Vázquez, R.; Cruz, A.; Muñoz, M. del C.; Túnez, I.; Muntané, J.; Padillo, F. J. Effects of Capecitabine and Celecoxib in Experimental Pancreatic Cancer. *Pancreatol. Off. J. Int. Assoc. Pancreatol. IAP Al* **2010**, *10* (5), 641–647. https://doi.org/10.1159/000288708.
(51) Qureshi, W. A.; Zhao, R.; Wang, H.; Ji, T.; Ding, Y.; Ihsan, A.; Mujeeb, A.; Nie, G.; Zhao, Y. Co-Delivery of Doxorubicin and Quercetin via MPEG–PLGA Copolymer Assembly for Synergistic Anti-Tumor Efficacy and Reducing Cardio-Toxicity. *Sci. Bull.* **2016**, *61* (21), 1689–1698. https://doi.org/10.1007/s11434-016-1182-z.
(52) Wang, Q.; Wieder, R. All-Trans Retinoic Acid Potentiates Taxotere-Induced Cell Death Mediated by Jun N-Terminal Kinase in Breast Cancer Cells. *Oncogene* **2004**, *23* (2), 426–433. https://doi.org/10.1038/sj.onc.1207040.
(53) Wang, Y.; He, Q.-Y.; Chen, H.; Chiu, J.-F. Synergistic Effects of Retinoic Acid and Tamoxifen on Human Breast Cancer Cells: Proteomic Characterization. *Exp. Cell Res.* **2007**, *313* (2), 357–368. https://doi.org/10.1016/j.yexcr.2006.10.016.
(54) Sun, R.; Liu, Y.; Li, S.-Y.; Shen, S.; Du, X.-J.; Xu, C.-F.; Cao, Z.-T.; Bao, Y.; Zhu, Y.-H.; Li, Y.-P.; Yang, X.-Z.; Wang, J. Co-Delivery of All-Trans-Retinoic Acid and Doxorubicin for Cancer Therapy with Synergistic Inhibition of Cancer Stem Cells. *Biomaterials* **2015**, *37*, 405–414. https://doi.org/10.1016/j.biomaterials.2014.10.018.
(55) Grunt, T.; Dittrich, E.; Offterdinger, M.; Schneider, S. M.; Dittrich, C.; Huber, H. Effects of Retinoic Acid and Fenretinide on the C-ErbB-2 Expression, Growth and Cisplatin Sensitivity of Breast Cancer Cells. *Br. J. Cancer* **1998**, *78* (1), 79–87. https://doi.org/10.1038/bjc.1998.446.
(56) Tian, Y.; Liu, G.; Wang, H.; Tian, Z.; Cai, Z.; Zhang, F.; Luo, Y.; Wang, S.; Guo, G.; Wang, X.; Powell, S.; Feng, Z. Valproic Acid Sensitizes Breast Cancer Cells to Hydroxyurea through Inhibiting RPA2 Hyperphosphorylation-Mediated DNA Repair Pathway. *DNA Repair* **2017**, *58*, 1–12. https://doi.org/10.1016/j.dnarep.2017.08.002.
(57) Hu, B.; Sun, D.; Sun, C.; Sun, Y.-F.; Sun, H.-X.; Zhu, Q.-F.; Yang, X.-R.; Gao, Y.-B.; Tang, W.-G.; Fan, J.; Maitra, A.; Anders, R. A.; Xu, Y. A Polymeric Nanoparticle Formulation of Curcumin in Combination with Sorafenib Synergistically Inhibits Tumor Growth and Metastasis in an Orthotopic Model of Human Hepatocellular Carcinoma. *Biochem. Biophys. Res. Commun.* **2015**, *468* (4), 525–532. https://doi.org/10.1016/j.bbrc.2015.10.031.
(58) Lopez Lopez, R.; van Rijswijk, R. E.; Wagstaff, J.; Pinedo, H. M.; Peters, G. J. The Synergistic and Antagonistic Effects of Cytotoxic and Biological Agents on the in Vitro Antitumour Effects of Suramin. *Eur. J. Cancer Oxf. Engl. 1990* **1994**, *30A* (10), 1545–1549. https://doi.org/10.1016/0959-8049(94)00285-d.
(59) Wu, X.; Huang, Z.; Liu, J.; Chen, Y.; Huang, H.; He, Y.; Li, D.; Zhang, L.; Du, Z.; Zhang, K.; Goodin, S.; Zheng, X. Effects and Mechanism of Inhibition of Naringin in Combination with Atorvastatin on Prostate Cancer Cells in Vitro and in Vivo. *Phytochem. Lett.* **2019**, *32*, 168–176. https://doi.org/10.1016/j.phytol.2019.06.008.
(60) Spingarn, A.; Sacks, P. G.; Kelley, D.; Dannenberg, A. J.; Schantz, S. P. Synergistic Effects of 13-Cis Retinoic Acid and Arachidonic Acid Cascade Inhibitors on Growth of Head and Neck Squamous Cell Carcinoma in Vitro. *Otolaryngol.--Head Neck Surg. Off. J. Am. Acad. Otolaryngol.-Head Neck Surg.* **1998**, *118* (2), 159–164. https://doi.org/10.1016/S0194-5998(98)80004-1.
(61) Scambia, G.; Ranelletti, F. O.; Benedetti Panici, P.; Piantelli, M.; Bonanno, G.; De Vincenzo, R.; Ferrandina, G.; Maggiano, N.; Capelli, A.; Mancuso, S. Inhibitory Effect of Quercetin on Primary Ovarian and Endometrial Cancers and Synergistic Activity with Cis-Diamminedichloroplatinum (II). *Gynecol. Oncol.* **1992**, *45* (1), 13–19. https://doi.org/10.1016/0090-8258(92)90484-z.

(62) Yabasin, I. B.; Ibrahim, M. M.; Adam, A.; Wilfred, S.-A.; Ziem, J. B.; Gao, P.; Kampo, S.; Qingping, W. Anticancer Effects of Vecuronium Bromide and Cisatracurium Besylate on Lung Cancer Cells (A549), in Vitro. *Biomed. Aging Pathol.* **2014**, *4* (4), 349–353. https://doi.org/10.1016/j.biomag.2014.07.004.
